# Elucidating the Antiviral Mechanism of Different MARCH Factors

**DOI:** 10.1128/mBio.03264-20

**Published:** 2021-03-02

**Authors:** Supawadee Umthong, Brian Lynch, Uddhav Timilsina, Brandon Waxman, Emily B. Ivey, Spyridon Stavrou

**Affiliations:** a Department of Microbiology and Immunology, Jacobs School of Medicine and Biomedical Sciences, University at Buffalo, Buffalo, New York, USA; Duke University Medical Center

**Keywords:** host restriction factors, retroviruses, envelope glycoproteins, human immunodeficiency virus, murine leukemia virus

## Abstract

The membrane-associated RING-CH (MARCH) proteins belong to a family of E3 ubiquitin ligases, whose main function is to remove transmembrane proteins from the plasma membrane. Recent work has shown that the human MARCH1, 2, and 8 are antiretroviral factors that target the human immunodeficiency virus type 1 (HIV-1) envelope glycoproteins by reducing their incorporation in the budding virions. Nevertheless, the dearth of information regarding the antiviral mechanism of this family of proteins necessitates further examination. In this study, using both the human MARCH proteins and their mouse homologues, we provide a comprehensive analysis of the antiretroviral mechanism of this family of proteins. Moreover, we show that human MARCH proteins restrict to various degrees the envelope glycoproteins of a diverse number of viruses. This report sheds light on the important antiviral function of MARCH proteins and their significance in cell intrinsic immunity.

## Abstract (precis)

This study examines the mechanism utilized by different MARCH proteins to restrict retrovirus infection. MARCH proteins block the incorporation of envelope glycoproteins to the budding virions.

## INTRODUCTION

The viral envelope is critical for viral replication and spread and thus a possible target for host antiviral factors. Such factors include the members of the membrane-associated RING-CH (MARCH) protein family, a unique family of E3 protein ligases, which target a number of immune receptors found in the plasma membrane (PM) (1). The human and mouse MARCH families consist of 11 members, of which 9 are transmembrane (TM) proteins with at least 2 TM domains (1). MARCH proteins have important regulatory roles in lymphocyte development (2–5) by targeting and removing from the PM a number of cellular membrane proteins including major histocompatibility complex II (MHC II) by MARCH1 and MARCH8 (4, 6–10) and discs large homologue 1 (DLG1) by MARCH2 (11). MARCH1 with MARCH8 and MARCH2 with MARCH3 are thought to form two homologous pairs, respectively, due to their structural homology, which suggests that they share similar substrates (1, 12, 13). MARCH1, 2, and 8 are expressed in the spleens, lymph nodes, and bone marrow (1, 2, 9, 13), tissues targeted by retroviruses, such as murine leukemia virus (MLV) and human immunodeficiency virus type 1 (HIV-1).

A common feature among all members of the MARCH protein family is the presence of a RING-CH (Really Interesting New Gene-CH) domain, which is a variant RING domain (4) and is critical for the ubiquitination and downregulation of their target proteins (12). Most MARCH proteins contain two or more TM domains that are essential for their localization and for MARCH-mediated target protein recognition (14–17). Additional domains have been identified in MARCH1 and MARCH8 to be important for their function including a domain in between the RING-CH domain and the transmembrane domain (DIRT domain), a short C-terminal sequence (VQNC), which is important for the proper folding of MARCH1 and MARCH8, and two C-terminal cytoplasmic tyrosine endocytic motifs (YXXΦ) (15, 18–22).

Immune receptors on the PM targeted by MARCH proteins are degraded in either the lysosome or the proteasome. MHC II, a target of MARCH1 and MARCH8, is degraded in the lysosome (4, 6, 23), while IL-1 receptor accessory protein (IL-1RAcP) is directed by MARCH8 for proteasomal degradation (24). Furthermore, the cytosolic tail of immune receptors (MHC II, CD86) targeted by MARCH proteins is critical for MARCH-mediated degradation (6, 19, 25, 26).

The retroviral envelope (*env*) gene encodes a surface-exposed glycoprotein that is essential for virus entry into a new cell (27). The extracellular subunit is referred to as the surface (SU) subunit (gp120 for HIV-1 and gp70 for MLV) and is responsible for receptor binding while the transmembrane (TM) subunit (gp41 for HIV-1 and p15E for MLV) is important for cell-virus fusion. The TM subunit of MLV gets further cleaved by the viral protease during or shortly after assembly, producing the p12E form of TM found in mature virions (28, 29). Recent reports identified human MARCH1, 2, and 8 as HIV-1 restriction factors, which reduce HIV-1 infectivity by blocking the incorporation of gp120 and gp41 in the viral envelope of newly synthesized virions by sequestering them intracellularly (30–32). Nevertheless, little is known about their mechanism of action, their conservation, and their breadth of antiviral function.

In this report, using both HIV-1 and MLV as well as human and mouse MARCH proteins, we found that unlike previous reports, HIV-1 and MLV envelope glycoproteins are not sequestered intracellularly but are degraded in the lysosome. In addition, we identified the domains of mouse March1 and 8 that are important for restriction and for physically binding to the retroviral envelope glycoproteins. Furthermore, using a variety of viral envelope glycoproteins, we demonstrated that human MARCH proteins have broad antiviral functions and target for degradation a variety of viral glycoproteins including those of lymphocytic choriomeningitis virus (LCMV), Lassa virus (LASV), and severe acute respiratory syndrome coronavirus 2 (SARS-CoV-2).

## RESULTS

### Transcriptional regulation of mouse *March* genes.

**FIG 1:**
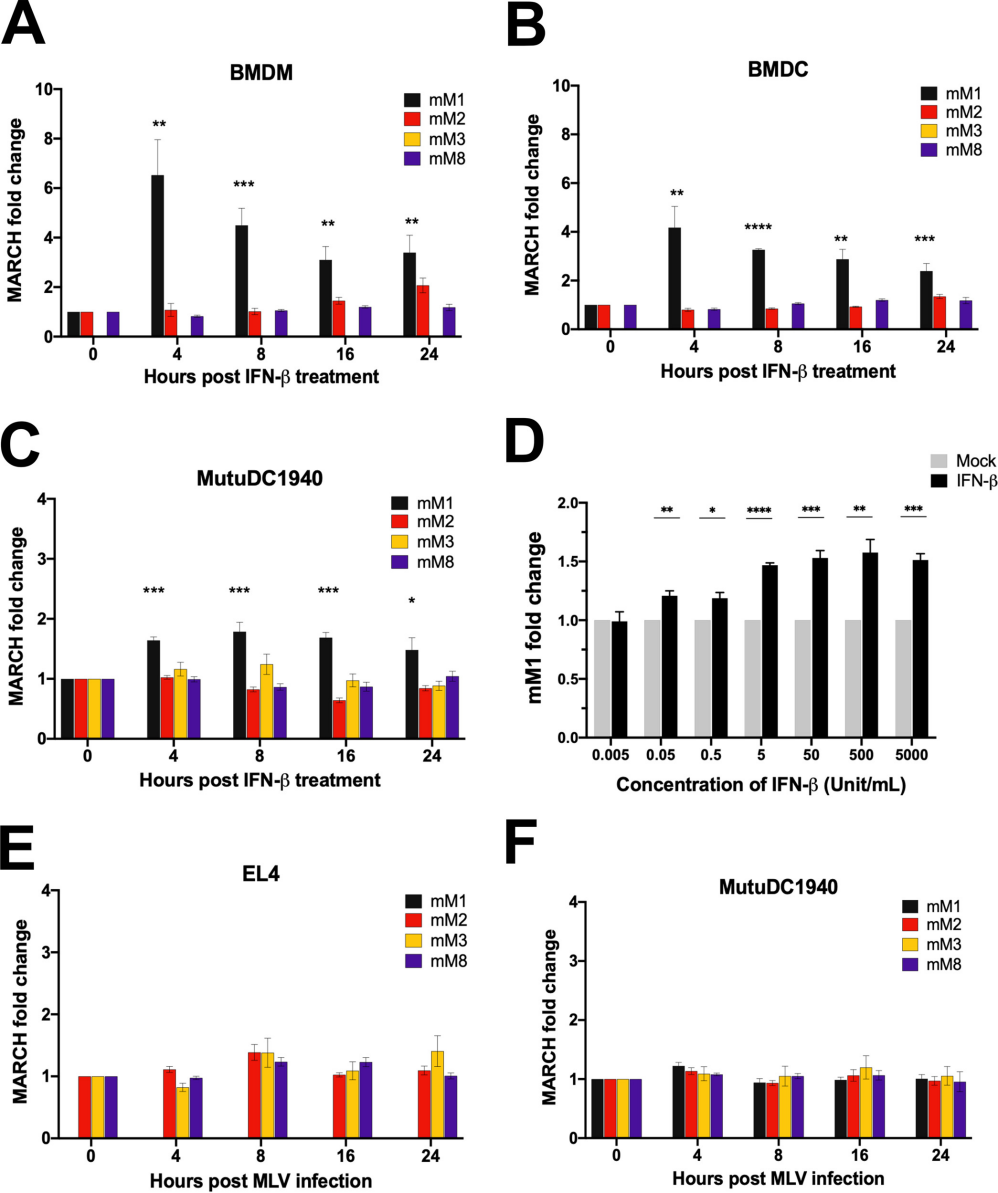
Expression levels of mouse *March1*, *2*, *3*, and *8* in the presence of IFN-β and MLV infection. (A to C) Fold expression changes of mouse *March1*, *2*, *3*, and *8* (*mM1*, *2*, *3*, and *8*) relative to untreated cells and normalized to GAPDH in (A) bone marrow-derived macrophages (BMDMs), (B) bone marrow-derived dendritic cells (BMDCs), and (C) MutuDC1940 cells at 0, 4, 8, 16, and 24 h after treatment with 500 units/ml of mouse IFN-β. Purity of BMDMs and BMDCs was determined by flow cytometry staining with anti-CD11b and anti-CD11c, respectively, as shown in Fig. S1D and E in the supplemental material. (D) Fold expression changes of mM1 relative to untreated cells and normalized to GAPDH after treatment of MutuDC1940 cells with different concentrations of IFN-β (0.005 to 5,000 units/ml) for 4 h. (E and F) Fold expression changes of mM1, 2, 3, and 8 relative to uninfected cells and normalized to GAPDH in (E) mouse T lymphocyte cell line EL4 and (F) MutuDC1940 cells at 4, 8, 16, and 24 h postinfection with MLV (5 MOI). Data shown represent averages of results from *n* = 3 independent experiments. All results are presented as means ± standard error of the mean (SEM). Statistical analysis performed using unpaired *t* test. *, *P* ≤ 0.05; **, *P* ≤ 0.01; ***, *P* ≤ 0.001; ****, *P* ≤ 0.001.

Human *MARCH1* and *MARCH2*, but not *MARCH8*, are type I interferon (IFN)-inducible genes (32). Thus, we examined the effect of murine IFN-β on the murine homologues of MARCH1, 2, and 8 in different primary and stable cell lines. We treated bone marrow-derived dendritic cells (BMDCs), bone marrow-derived macrophages (BMDMs), EL4 (murine T lymphocyte cell line), NIH 3T3 (murine fibroblast cell line), and MutuDC1940 (immortalized mouse dendritic cell [DC] line [33]) with murine IFN-β (500 U/ml). We collected RNA at different time points and performed RT-PCR to determine mouse *March1*, *2*, *3*, and *8* expression levels. We found that mouse *March1* was expressed only in MutuDC1940, BMDCs, and BMDMs while mouse *March2* and *March8* were expressed in all cell lines tested (Fig. 1A to C and also Fig. S1A and B in the supplemental material). Mouse *March3* was expressed only in transformed cell lines (Fig. 1C and Fig. S1A to C) and not in primary cells (Fig. 1A and B). Interestingly, only mouse *March1* was IFN-β inducible in all cell lines expressing it (BMDMs, BMDCs, and MutuDC1940) (Fig. 1A to C). Mouse *March3*, *8*, and *2*, unlike human *MARCH2*, were not IFN-β inducible (Fig. 1A to C and Fig. S1A and B). By treating MutuDC1940 with various amounts of murine IFN-β, we found that IFN-β upregulated mouse *March1* mRNA levels in a dose-dependent manner (Fig. 1D). To determine the effect of MLV infection on the expression levels of mouse *March1*, *2*, *3*, and *8*, we infected MutuDC1940, EL4, and NIH 3T3 with MLV (multiplicity of infection [MOI] of 5) and at different time points collected RNA and performed RT-PCR to determine their expression levels. We found that MLV infection had no effect on mouse *March1*, *2*, *3*, and *8* expression in all cell lines tested (Fig. 1E and F and Fig. S1C). Thus, we concluded that MLV infection has no effect on mouse *March1*, *2*, *3*, and *8* transcript levels and only mouse *March1* is an IFN-stimulated gene.

10.1128/mBio.03264-20.1 FIG S1 Expression levels of mouse *March1, 2, 3*, and *8* in the presence of IFN-β and MLV infection in EL4 and NIH 3T3 cells. (A and B) Fold expression changes of mouse *March1, 2, 3*, and *8* (*mM1, 2, 3*, and *8*) relative to untreated cells and normalized to GAPDH in (A) EL4 mouse T cell line and (B) NIH 3T3 mouse fibroblasts treated with 500 units/ml of mouse IFN-β and harvested at 0, 4, 8, 16, and 24 h posttreatment. (C) Fold expression changes of mM1, 2, 3, and 8 relative to uninfected cells and normalized to GAPDH in NIH 3T3 cells at 4, 8, 16, and 24 h after infection with MLV (5 MOI). (D) Purity of bone marrow-derived macrophages (BMDMs) and (E) bone marrow-derived dendritic cells (BMDCs) used in Fig. 1 was confirmed by staining with either (D) the macrophage cell surface antibody CD11b-FITC or (E) the dendritic cell surface antibody CD11c-APC. Data shown represent averages of results from *n* = 3 independent experiments. All results are presented as means ± standard errors of the means (SEM). Statistical analysis performed using unpaired *t* test. **, *P* ≤ 0.01; ***, *P* ≤ 0.001. Download FIG S1, TIF file, 2.1 MB. Copyright © 2021 Umthong et al. 2021 Umthong et al. https://creativecommons.org/licenses/by/4.0/ This content is distributed under the terms of the Creative Commons Attribution 4.0 International license.

### Mouse March1 and 8 do not sequester the MLV and MMTV envelope glycoproteins intracellularly but target them for degradation.

**FIG 2:**
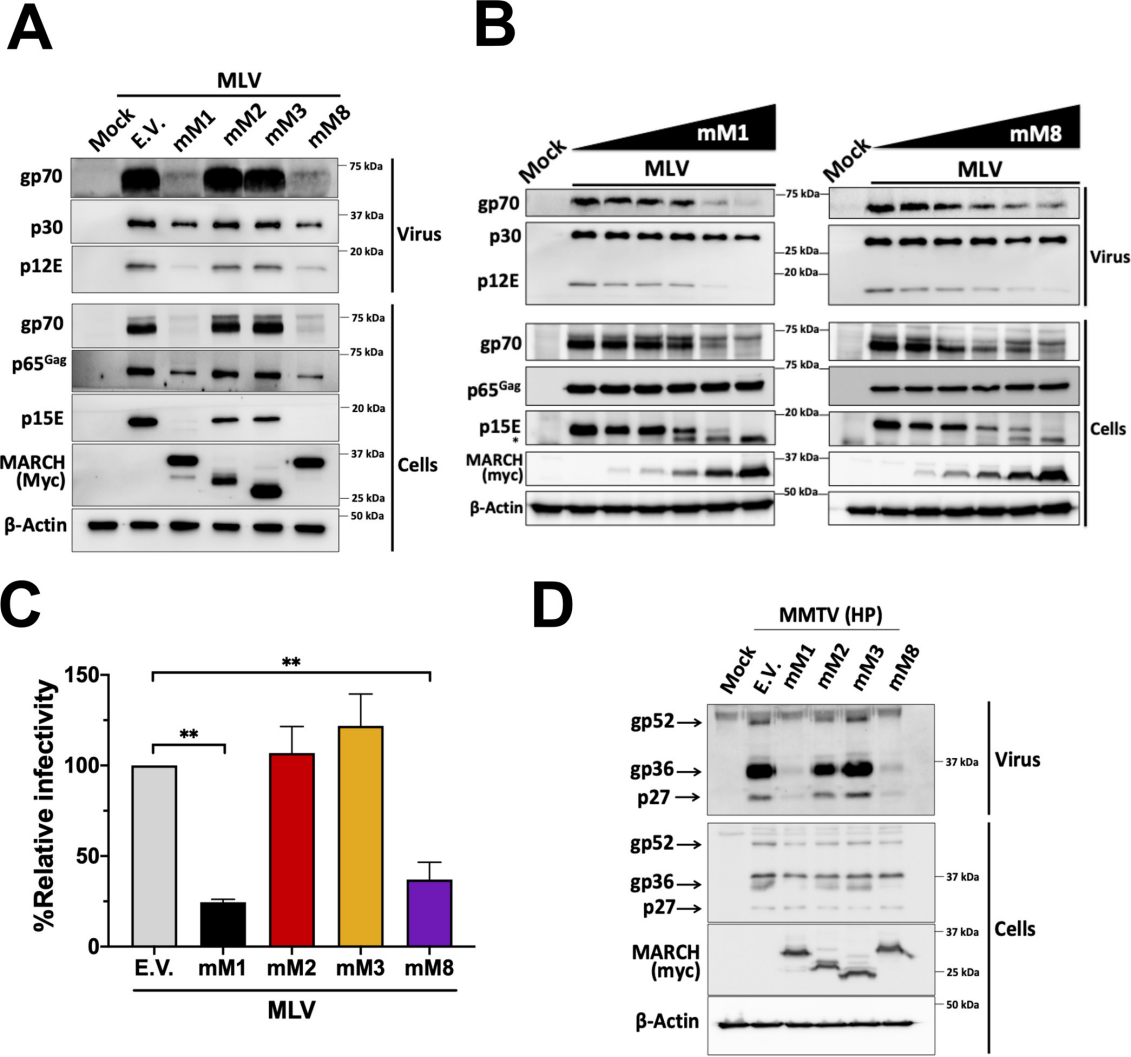
Mouse March1 and mouse March8 block retrovirus infection by targeting the retroviral envelope glycoproteins. (A) Mouse March1 (mM1) and mM8 block the incorporation of the MLV envelope glycoproteins (p15E and gp70) in nascent virions by targeting them for degradation. (B) mM1 and mM8 target the MLV envelope glycoproteins for degradation in a dose-dependent manner. For both panels A and B, 293T cells were cotransfected with an MLV molecular clone and the indicated mouse *March* constructs. At 48 h posttransfection, cells and released virus in the culture medium were harvested, and the indicated proteins were analyzed by immunoblotting using anti-MLV p30 (detects both p30 and p65^Gag^), anti-MLV gp70, anti-MLV p15E/p12E, anti-myc (for detection of mM1, 2, 3, and 8), and anti-β-actin antibodies. (C) Virus produced in the presence of mM1 and mM8 has decreased infectivity. NIH 3T3 cells were infected with equal amounts of 293T-derived MLV-luciferase reporter virus produced in the presence of mM1, 2, 3, or 8 or empty vector (E.V.). Cells were harvested 24 h postinfection, and luciferase levels were measured. The percentage (%) of relative infectivity was determined with respect to virus produced in the presence of E.V. All results are presented as means ± SD. Statistical significance was determined by one-way ANOVA. **, *P* < 0.01. (D) mM1 and mM8 target MMTV gp36 for degradation. 293T cells were cotransfected with an infectious genetically engineered MMTV hybrid provirus (HP) and either mM1, 2, 3, 8, or E.V. At 48 h posttransfection, cells and released virus in the culture medium were harvested, and the indicated proteins were analyzed by immunoblotting using anti-MMTV, anti-myc (for detection of mM1, 2, 3, and 8), and anti-β-actin antibodies. Results are shown for *n* = 3 independent experiments. Representative immunoblotting results are shown in panels A, B, and D.

To determine the effect that mouse MARCH genes have on retrovirus envelope glycoproteins, we cotransfected 293T cells with an MLV molecular clone and with either an empty vector (E.V.) or mouse *March1*, *2*, *3*, or *8*. Cells and the media were harvested 48 h posttransfection followed by Western blot assays to determine the levels of the MLV envelope glycoproteins (gp70/SU and p15E/p12E/TM). In the cell fractions, we found that the protein levels of both gp70 and p15E were reduced in the presence of mouse March1 and mouse March8, while mouse March2 and 3 had no effect (Fig. 2A). Furthermore, virions produced in the presence of mouse March1 or 8 had significantly lower levels of gp70 and p12E than virions produced in cells transfected with either E.V. or mouse *March2* or *3* (Fig. 2A). By using various levels of mouse March1 and mouse March8, we found that mouse March1 and 8 degraded gp70 and p15E in a dose-dependent manner (Fig. 2B). Interestingly, when probing for p15E in the cell fractions, we observed a band (shown with an asterisk) migrating slightly faster than p15E when using larger amounts of either mouse March1 or mouse March8, which probably reflects a degradation intermediate of p15E (Fig. 2B). To determine if the decreased incorporation of gp70 and p15E in the nascent virions affected virus infectivity, we produced MLV-luciferase reporter viruses in the presence of either mouse *March1*, *2*, *3*, or *8* or E.V. NIH 3T3 cells were infected with equal amounts of MLV CA, and luminescence was measured 48 h postinfection. We found that virions produced in the presence of mouse March1 or 8 were significantly less infectious than those produced in the presence of mouse March2 or 3 (Fig. 2C). We also investigated the effect of mouse March1, 2, 3, and 8 on the viral envelope glycoproteins of another murine retrovirus, mouse mammary tumor virus (MMTV). We cotransfected 293T cells with a plasmid encoding an infectious genetically engineered MMTV hybrid provirus (HP) (34) and either mouse *March1*, *2*, *3*, or *8* or E.V. Cell and medium fractions were harvested, and Western blot assays were performed to determine the levels of the MMTV envelope glycoproteins and capsid (gp52/SU, gp36/TM, and p27/CA). Interestingly, while mouse March1 and mouse March8 abrogated the cellular levels of gp36, they had no effect on the gp52 and p27 cellular levels of MMTV (Fig. 2D). When examining the virus fraction, we found that virus levels were abrogated in the cells transfected with mouse *March1* and mouse *March8*, while mouse *March2* and *3* had no effect (Fig. 2D). Interestingly, in our MMTV experiments we do not even detect CA/p27 in the virus fraction (whereas the cellular levels of CA/p27 are unaffected) when mouse March1 and mouse March8 are present (Fig. 2D), which is different from what we observed with MLV, where in the presence of mouse March1 and 8 we still detected CA/p30 levels in the virus fraction, suggesting the presence of virions either lacking or having only very low levels of viral envelope glycoproteins incorporated (Fig. 2A), similar to what was seen with HIV-1 (30–32). It is possible that the differences in the CA levels in the virus fractions between MLV and MMTV (bald virus versus no virus) may be attributed to differences in the mechanisms and sites of assembly and release between these two murine retroviruses (35, 36).

### Human and mouse MARCH proteins differ in their abilities to degrade retroviral envelope glycoproteins.

**FIG 3:**
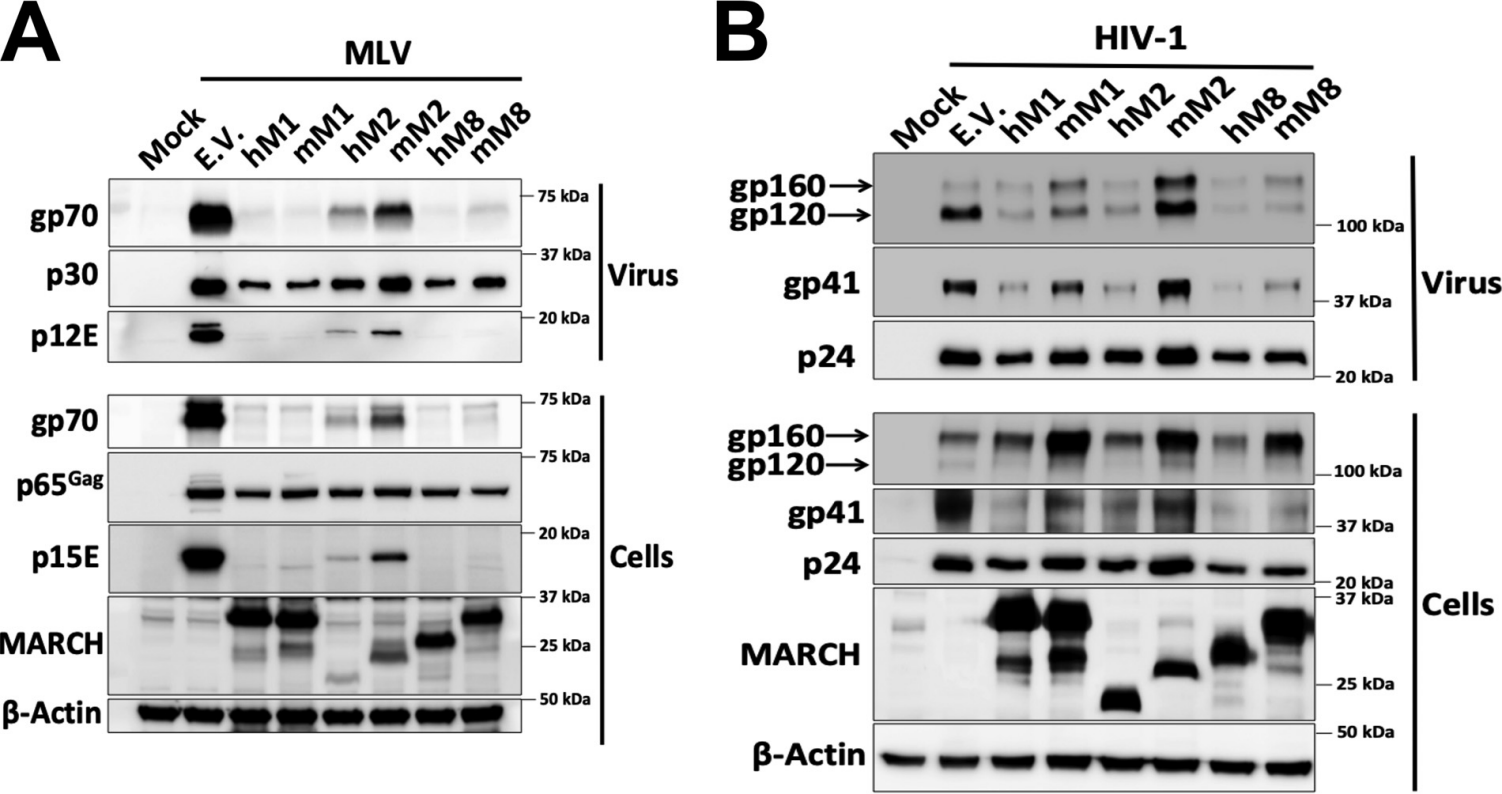
Mouse March and human MARCH proteins differ in their ability to target retroviral envelope glycoproteins. (A) MLV envelope glycoproteins are targeted for degradation by mouse March (mM1) and mM8 and human MARCH (hM1), hM2, and hM8. (B) HIV-1 envelope glycoproteins are targeted for degradation by mM8 and hM1, 2, and 8. For both panels A and B, 293T cells were cotransfected with (A) MLV or (B) HIV-1 infectious clone and *hmM1*, *2*, or *8*. At 48 h posttransfection, cells and released virus in the culture medium were harvested, and the indicated proteins were analyzed by immunoblotting using anti-MLV p30 (detects both p30 and p65^Gag^), anti-MLV gp70, anti-MLV p15E/p12E, anti-HIV-1 envelope (detects both gp120 and gp160), anti-gp41, anti-p24, anti-myc (for detection of mM1, 2, and 8), anti-MARCH1, 2, 8 and anti-β-actin antibodies. Results are shown for *n* = 3 independent experiments. Representative immunoblotting results are shown for panels A and B.

In contrast to previous studies, our aforementioned data show that the cellular levels of the MLV envelope glycoproteins (gp70 and p15E) are reduced in the presence of mouse March1 and 8, suggesting that the viral envelope glycoproteins are degraded and not sequestered intracellularly. We hypothesized that the reason for this discrepancy is due to the difference in the species of origin for the MARCH proteins we used in our system (mouse) versus those in previous reports (human). To investigate this, we cotransfected 293T cells with an MLV molecular clone and with either the human or mouse *MARCH1*, *2*, or *8* or an E.V. We observed that both human and mouse MARCH1 and 8 abrogated the cellular levels of gp70 and p15E (Fig. 3A). Interestingly, human MARCH2, and to a lesser extent mouse March2, reduced the gp70 and p15E levels in the cellular and viral fractions (Fig. 3A). We thus concluded that mouse and human MARCH1 and 8 as well as human MARCH2 degrade and do not sequester intracellularly the MLV envelope glycoproteins.

We then speculated that HIV-1 envelope glycoprotein intracellular sequestration by the human MARCH proteins might be species specific. Therefore, we examined the effect of human and mouse MARCH1, 2, and 8 on HIV-1. 293T cells were cotransfected with NL4-3 (an HIV-1 lab strain) and either human or mouse *MARCH1*, *2*, and *8* or an E.V. We found that all human MARCH proteins (MARCH1, 2, and 8) decreased the cellular levels of both HIV-1 envelope glycoproteins, gp120 and gp41 (Fig. 3B). On the other hand, only mouse March8 markedly decreased the gp120 and gp41 cellular levels (Fig. 3B). Mouse March2, similar to what we observed with MLV, had no effect (Fig. 3B). Mouse March1, whereas it potently restricted the MLV envelope glycoproteins, partially decreased the levels of gp120 and gp41 in the budding virions (Fig. 3B). In summary, our data show that while all human MARCH proteins tested are potent restrictors of retroviral envelope glycoproteins, of the mouse March homologues only mouse March8 was able to potently restrict both MLV and HIV-1 envelope glycoproteins. Finally, in contrast to previous reports, we found that MARCH proteins, both the human and mouse homologues, cause degradation and not intracellular sequestration of the retroviral envelope glycoproteins.

### Mapping the regions of mouse March1 and mouse March8 responsible for their antiviral function.

**FIG 4:**
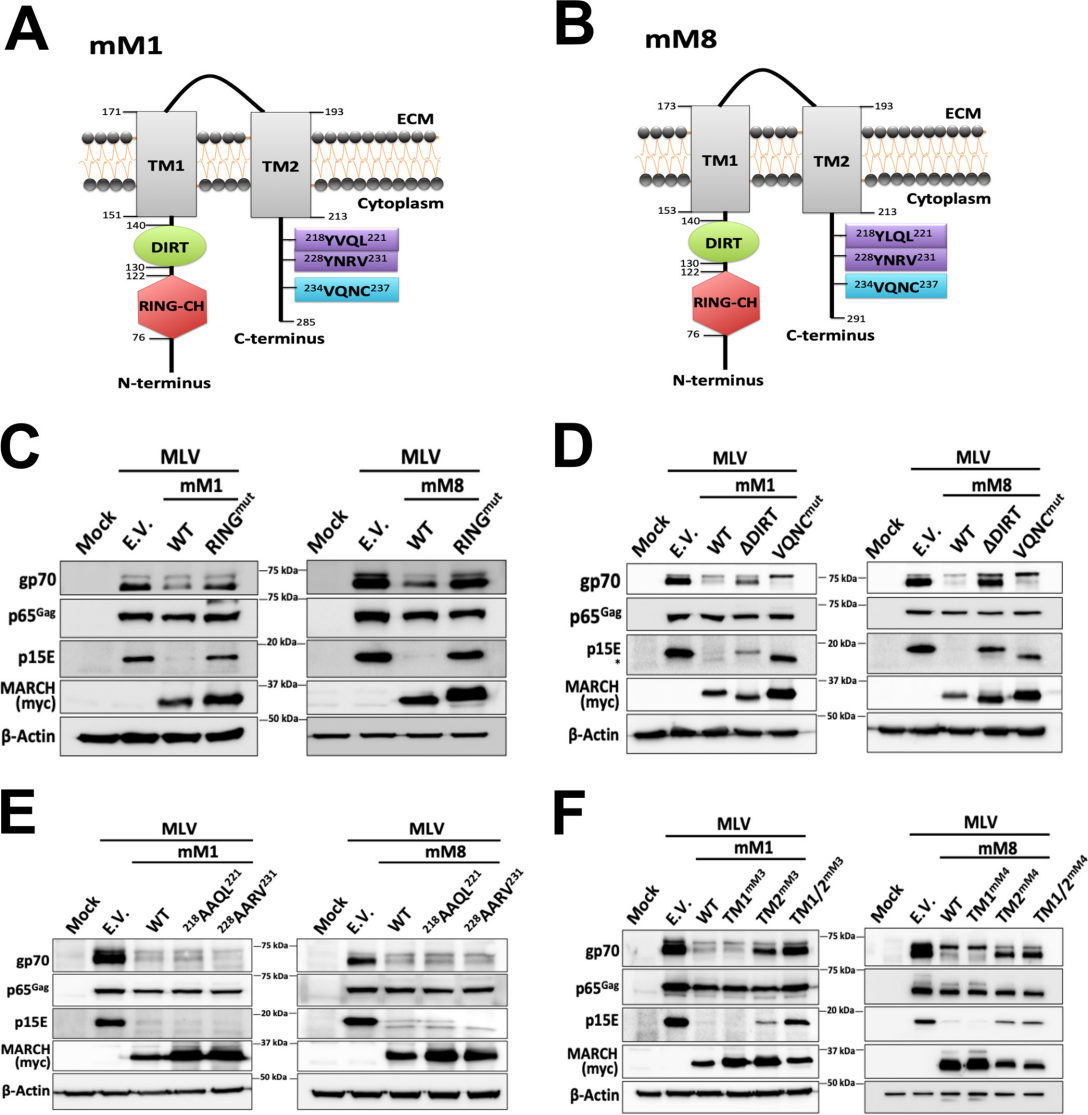
The role of different mouse March1 and mouse March8 domains on retroviral envelope glycoprotein restriction. (A) Schematic diagram of mouse March (mM1) and (B) mM8 and their respective domains: Really Interesting New Gene-CH (RING-CH) domain, the domain in between RING-CH domain and transmembrane domain (DIRT), N-terminal and C-terminal TM domains (TM1 and TM2, respectively), two tyrosine endocytic motifs, and a VQNC motif. (C to F) Only the RING-CH, DIRT, and TM2 domains of mM1 and 8 are important for the degradation of MLV envelope glycoprotein. 293T cells were cotransfected with either (C) RING-CH mutant (RING^mut^) *mM1* and *mM8*, (D) *mM1* and *mM8* with the DIRT domain deleted (ΔDIRT) or the VQNC domain mutated (VQNC^mut^), (E) *mM1* or *mM8* with mutations in the tyrosine endocytic motifs and an MLV infectious clone, or (F) *mM1* or *mM8* with either the N-terminal (TM1), C-terminal (TM2), or both TM domains (TM1/2) swapped with those of *mM3* (in the case of *mM1*) and *mM4* (in the case of *mM8*). Cells were harvested 48 h posttransfection, and lysates were analyzed by immunoblotting using anti-MLV p30 (detects p65^Gag^), anti-MLV gp70, anti-MLV p15E, anti-myc (for detection of the mM1 and 8 proteins), and anti-β-actin antibodies. Results are shown for *n* = 3 independent experiments. Representative immunoblotting results are shown for panels C through F.

Mouse March1 and 8, due to their homology, have sequence and structural similarities (1, 4, 9). Both mouse and human MARCH1 and 8 have a variant RING domain (RING-CH) that is located in the N-terminal cytoplasmic tail (Fig. 4A and B) and is essential for their function (1, 30–32), a DIRT domain (18), a VQNC sequence (15, 19, 20, 22), two tyrosine (Y) motifs (YXXΦ) present in the C-terminal cytoplasmic tail (15, 21), and two TM domains (14) (Fig. 4A and B). To determine the importance of these domains in retrovirus restriction, we generated a series of mouse March1 and March8 constructs with mutations or deletions in the various domains. We first verified that the mutant mouse March1 and March8 constructs continued to localize in cellular membranes. In the case of mouse March1, we transfected 293T cells with the wild-type and mutant March1 constructs and then stained the cell surface of the transfected cells with an anti-March1 antibody followed by flow cytometry and found that all mouse March1 constructs localize on the plasma membrane (Fig. S2A). Unfortunately, there was no suitable mouse March8 antibody available for flow cytometry. Thus, we transfected 293T cells and isolated membrane-bound proteins followed by Western blot assays probing for mouse March8 and for glyceraldehyde-3-phosphate dehydrogenase (GAPDH) to verify the purity of our membrane fractions. We found all mutant March8 constructs localized to cellular membranes similarly to wild-type March8 (Fig. S2B and C). Subsequently, we cotransfected 293T cells with an MLV molecular clone and the various mouse March1 and mouse March8 mutant constructs followed by Western blotting to detect any changes in the gp70 and p15E protein levels. When we introduced mutations in the RING-CH domains of either mouse March1 or mouse March8, we found that the levels of gp70 and p15E were similar to those seen when we transfected with E.V. (Fig. 4C). Furthermore, deletion of the mouse March8 DIRT domain (ΔDIRT) rescued gp70 and p15E from March8-mediated degradation (Fig. 4D, left panel); however, deletion of the mouse March1 DIRT domain (ΔDIRT) had only a partial effect (Fig. 4D, right panel). Mutations in the VQNC motif (mouse March1/VQNC^mut^ and March8/VQNC^mut^) or the two YXXΦ endocytic motifs had no effect on March-mediated degradation of gp70 and p15E (Fig. 4D and E). Similarly to Fig. 2B, we observed a similar band (shown with an asterisk) migrating slightly faster than p15E, which probably reflects a degradation intermediate (Fig. 4D). To examine the role of the TM domains, we independently swapped the TM domains of mouse March1 with those of mouse March3, as mouse March3 has no antiretroviral function. We found that only the second TM domain was important for its antiretroviral activity (Fig. 4F, left panel). We initially exchanged the March8 TM domains with those of mouse March3, but it affected protein expression (data not shown). We thus swapped the TM domains of mouse March8 with those of mouse March4, as mouse March4 has no antiretroviral effect (Fig. S3A). We observed that similarly to mouse March1, swapping the second TM domain of mouse March8 with that of mouse March4 rendered mouse March8 unable to restrict gp70 and p15E (Fig. 4F, right panel). Our findings show that only the RING-CH, DIRT, and the second TM domain of mouse March1 and March8 are critical for retroviral envelope glycoprotein restriction.

10.1128/mBio.03264-20.2 FIG S2 Mouse March1 and mouse March8 localize in cellular membranes. (A) Wild-type (WT) mouse March1 (mM1) and mM1 mutants (RING^mut^, ΔDIRT, TM1^mM3^, TM2^mM3^, TM1/2^mM3^, ^218^AAQL^221^, ^228^AARV^231^, and VQNC^mut^) localize in the plasma membrane of the transfected cells. 293T cells were transfected with *mM1* constructs used in Fig. 4 and 6. Cells were harvested and surface stained with anti-MARCH1 or rabbit IgG isotype control followed by anti-rabbit IgG (Alexa Fluor 647) and analyzed by flow cytometry. Histogram plots indicate percentage of cells expressing mM1 on the cell membrane. (B and C) Wild-type (WT) mouse March8 (mM8) and mM8 mutants (RING^mut^, ΔDIRT, TM1^mM4^, TM2^mM4^, TM1/2^mM4^, ^218^AAQL^221^, ^228^AARV^231^, and VQNC^mut^) localize in the cellular membranes of the transfected cells. 293T cells were transfected with the various *mM8* constructs used in Fig. 4 and 6. Cells were harvested, and integral membrane proteins were extracted using the MEM-PER Plus membrane extraction kit (Thermo Scientific) followed by Western blot assays using anti-myc (mM8 detection) and anti-GAPDH (marker for purity of the membrane fractions). For empty vector (E.V.) and mM8 WT transfection, we provide the membrane fraction (M) and cellular fraction (C). Shown are the results of a single experiment (representative of two independent experiments). Download FIG S2, TIF file, 2.5 MB. Copyright © 2021 Umthong et al. 2021 Umthong et al. https://creativecommons.org/licenses/by/4.0/ This content is distributed under the terms of the Creative Commons Attribution 4.0 International license.

10.1128/mBio.03264-20.3 FIG S3 Mouse Macrh3 and mouse March4 do not inhibit or physically interact with MLV p15E. (A) Mouse March4 does not target MLV envelope proteins for degradation. 293T cells were cotransfected with an MLV infectious clone and mouse *March4* (*mM4*). At 48 h posttransfection, cells and released virus in the culture medium were harvested, and the indicated proteins were analyzed by immunoblotting using anti-MLV p30 (detects p30 and p65^Gag^), anti-MLV gp70, anti-MLV p15E/p12E, anti-myc (for detection of mM4), and anti-β-actin antibodies. Shown are the results of a single experiment (representative of three independent experiments). (B) Mouse March3 and (C) Mouse March4 do not interact with MLV p15E. 293T cells were cotransfected with an MLV infectious clone and either (B) *mM1* or *mM3* or (C) *mM8* or *mM4* and were harvested 48 h posttransfection. Cell lysates were immunoprecipitated with anti-myc (mM1, mM3, mM8, and mM4) followed by Western blot assays probing with anti-myc (mM1, mM3, mM8, and mM4) or anti-15E. Shown are the results of a single experiment (representative of three independent experiments). Download FIG S3, TIF file, 3.0 MB. Copyright © 2021 Umthong et al. 2021 Umthong et al. https://creativecommons.org/licenses/by/4.0/ This content is distributed under the terms of the Creative Commons Attribution 4.0 International license.

### The cytosolic tail of the MLV envelope glycoprotein is crucial for MARCH-mediated restriction.

**FIG 5:**
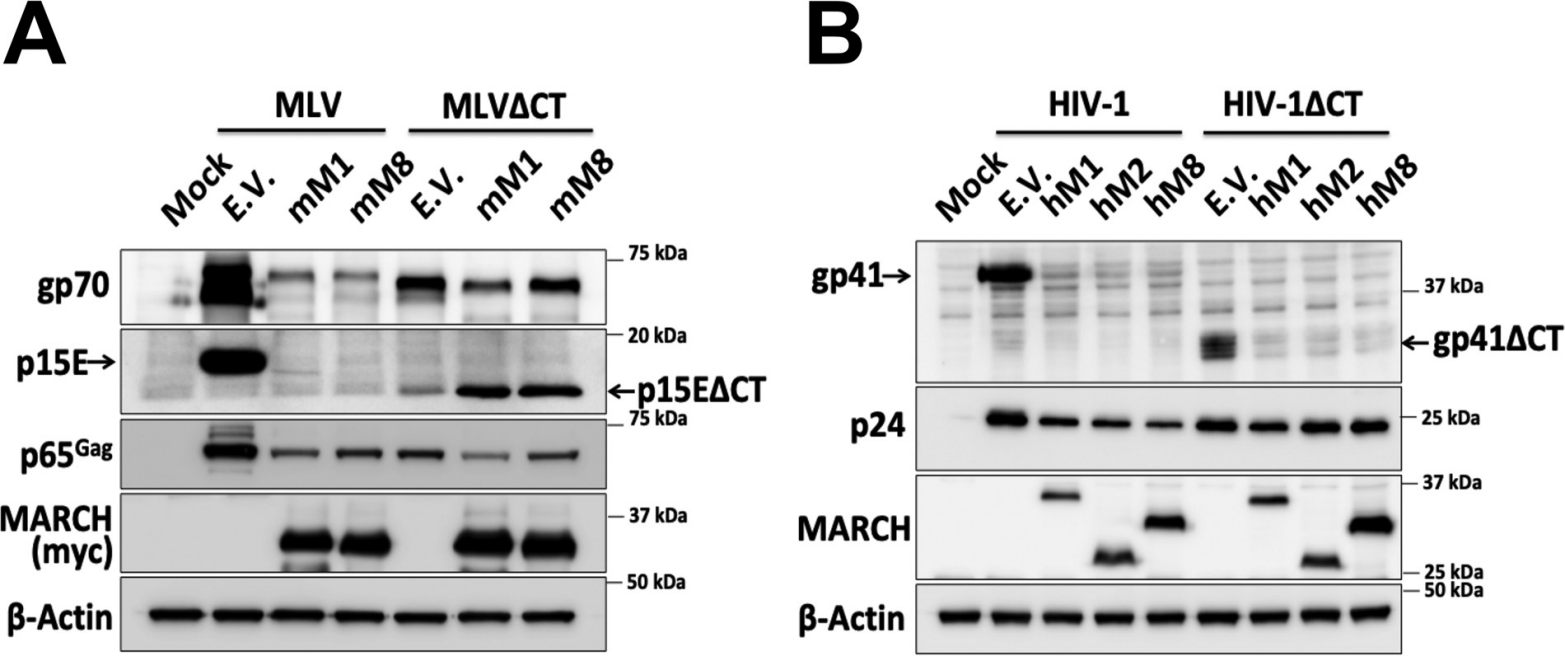
The MLV envelope glycoprotein cytoplasmic tail is critical for MARCH-mediated restriction, but the HIV envelope cytoplasmic tail is dispensable. 293T cells were cotransfected in panel A with wild-type MLV or MLV with the cytosolic tail deleted (MLVΔCT) along with either mouse March1 (mM1), mM2, or mM8 and in panel B with wild-type HIV or HIV with the cytosolic tail deleted (HIVΔCT) along with either human MARCH1 (hM1), hM2, or hM8. For panels A and B, at 48 h posttransfection cells were harvested and lysates were analyzed by immunoblotting using anti-MLV p30 (detects p65^Gag^), anti-MLV gp70, anti-MLV p15E, anti-myc (for detection of human and mouse MARCH proteins), anti-gp41, anti-p24, and anti-β-actin antibodies. Results are shown for *n* = 3 independent experiments. Representative immunoblotting results are shown for panels A and B.

Previous studies have shown that the cytoplasmic tails of the immune receptors targeted by MARCH proteins (e.g., MHC II) are critical for their removal from the cell surface (6, 15, 19, 26, 37). The MLV p15E contains a C-terminal cytosolic tail that is 35 amino acids long (38). To determine if the cytoplasmic tail of p15E is critical for its MARCH-mediated removal from the PM, we created an MLV molecular clone that did not express the cytoplasmic tail of p15E (pMLVΔCT) by introducing a stop codon at the N terminus of the p15E cytoplasmic tail region. We cotransfected cells with an MLV infectious clone expressing either an intact p15E cytoplasmic tail (pMLV) or pMLVΔCT in the presence of either mouse March1, mouse March8, or E.V. We found that gp70 and p15E with an intact cytoplasmic tail were degraded in the presence of either mouse March1 or 8. On the other hand, when p15E lacked the cytoplasmic tail, gp70 and p15EΔCT were resistant to the deleterious effect of mouse March1 and 8 (Fig. 5A). Paradoxically, we repeatedly noticed that p15E lacking the cytoplasmic tail was detected in Western blots at higher levels in the presence of either March1 or March8 compared to E.V. (Fig. 5A), the reason for which is unclear.

The retroviral envelope glycoprotein cytoplasmic tail ranges in size, with the cytoplasmic tail of lentiviruses being the longest (27). Due to the differences in size and composition between the MLV p15E and HIV-1 gp41 cytoplasmic tails, it is possible that unlike the MLV p15E cytoplasmic tail, the HIV-1 gp41 cytoplasmic tail is dispensable for MARCH-mediated restriction. Therefore, we generated an HIV-1 molecular clone that does not express the cytoplasmic tail of gp41 (NL4-3ΔCT) by introducing two stop codons at the N terminus of the gp41 cytoplasmic tail. We cotransfected 293T cells with either NL4-3 with intact gp41 cytoplasmic tail (HIV-1) or NL4-3ΔCT (HIV-1ΔCT) and either human *MARCH1*, *2*, *8*, or E.V. Unlike our findings with MLV, we noticed that the gp41 cytoplasmic tail is dispensable for MARCH-mediated restriction (Fig. 5B). In conclusion, the difference in the importance of the cytoplasmic tail between gp41 and p15E for MARCH-mediated restriction suggests that MARCH proteins may use different mechanisms to restrict MLV and HIV-1.

### Mouse March1 and 8 physically interact with the p15E subunit of the MLV envelope utilizing different transmembrane domains.

**FIG 6:**
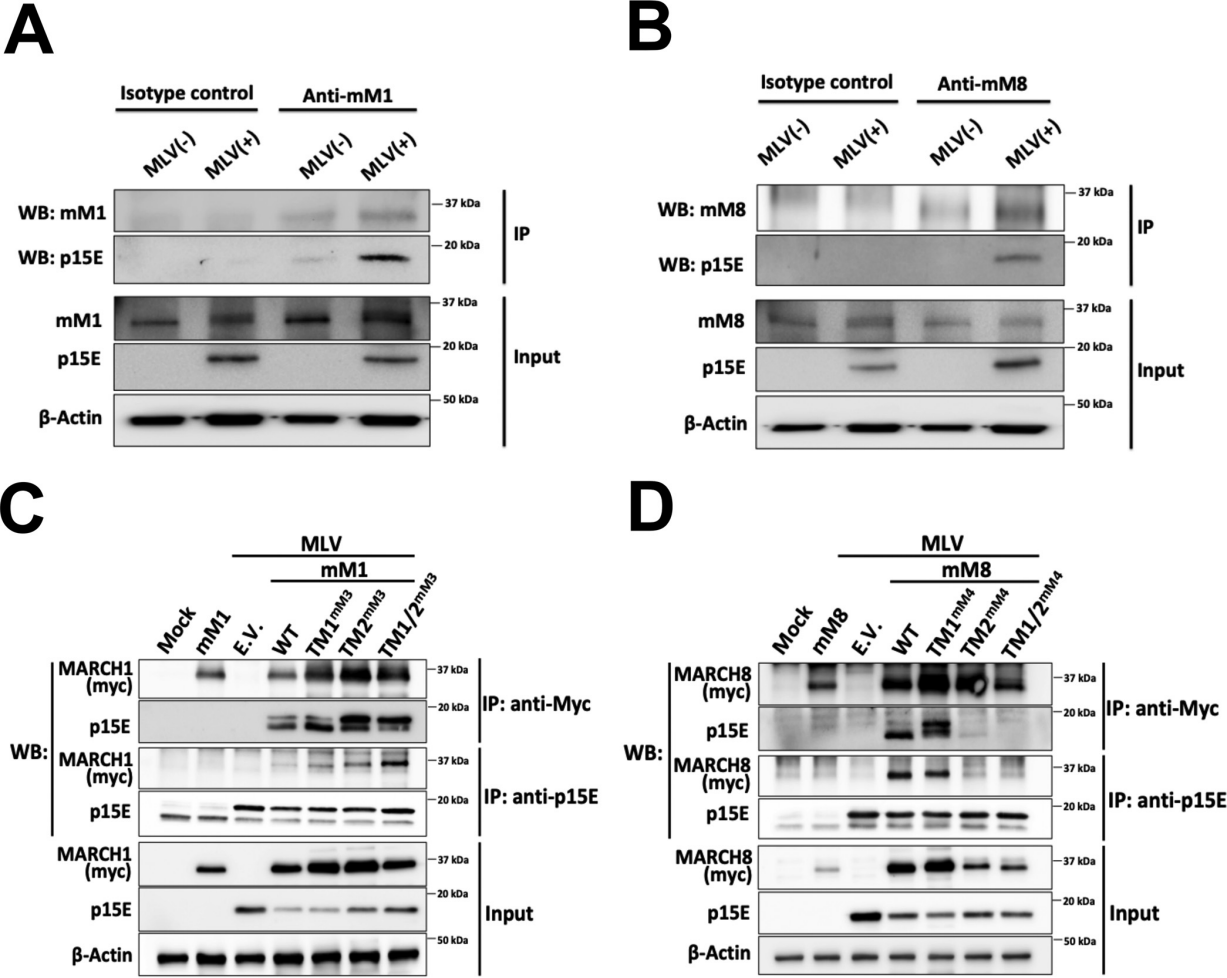
Mouse March1 and mouse March8 physically interact with MLV p15E. (A and B) MutuDC1940 cells, which express endogenous levels of both mouse March1 (mM1) and mM8, were infected with MLV (10 MOI). Cells were harvested 72 h postinfection, and lysates were immunoprecipitated with (A and B) an isotype control, (A) anti-MARCH1, and (B) anti-MARCH8 and analyzed in Western blots (WB) with anti-p15E, anti-MARCH1, anti-MARCH8, and anti-β-actin. (C and D) 293T cells were cotransfected with an MLV infectious clone and either (C) mouse *March1* (*mM1*) or (D) *mM8* and their TM mutants used in Fig. 4. Cells were harvested 48 h posttransfection, and lysates were immunoprecipitated with anti-myc (mM1 and mM8) and anti-p15E followed by Western blot analyses probing with anti-p15E, anti-myc (mM1 and mM8), and anti-β-actin antibodies. Results are shown for *n* = 3 independent experiments. Representative immunoblotting results are shown for panels A through D.

MARCH proteins physically interact with their cellular targets (MHC II, IL-1RAcP, etc.) (24, 39). To determine if endogenous mouse March1 and mouse March8 directly interacted with MLV p15E, we infected MutuDC1940 cells, which express endogenous levels of mouse *March1* and mouse *March8* (Fig. 1C), with MLV. Cells were lysed, and coimmunoprecipitations (coIPs) were performed by immunoprecipitating either an isotype control, mouse March1, or mouse March8. A p15E-specific antibody revealed that MLV p15E coimmunoprecipitated with endogenous mouse March1 (Fig. 6A) and March8 (Fig. 6B). To determine the importance of the TM domains of mouse March1 for binding to MLV p15E, we transfected 293T cells with mouse *March1*; mouse *March1* with the TM domains swapped with those from *March3*, as mouse March3 does not bind to p15E (Fig. S3B); and an MLV molecular clone. We performed coIPs with anti-myc (March1) or anti-p15E. We found that March1 coimmunoprecipitated with MLV p15E independently of the TM domains present (Fig. 6C). MLV p15E also coimmunoprecipitated with wild-type March1 as well as March1 containing the March3 TM domains (Fig. 6C). Thus, we concluded that mouse March1 interacts with p15E independently of the TM domains. To determine the role of the TM domains of mouse March8 on its interaction with MLV p15E, we used the mouse March8 constructs with the mouse March4 TM domains mentioned above (Fig. 4F). March4 does not bind to p15E (Fig. S3C). We found that changing the C-terminal TM domain (TM2) of mouse March8 to that of mouse March4 abolished the mouse March8-p15E interaction (Fig. 6D). In summary, our findings show that mouse March1 and mouse March8 physically interact with the MLV p15E, and while for mouse MARCH1, its interaction with p15E is TM independent, mouse MARCH8 interacts with p15E via the C-terminal TM domain (TM2).

### Mouse March1 and 8 target the MLV envelope glycoprotein for lysosomal degradation.

**FIG 7:**
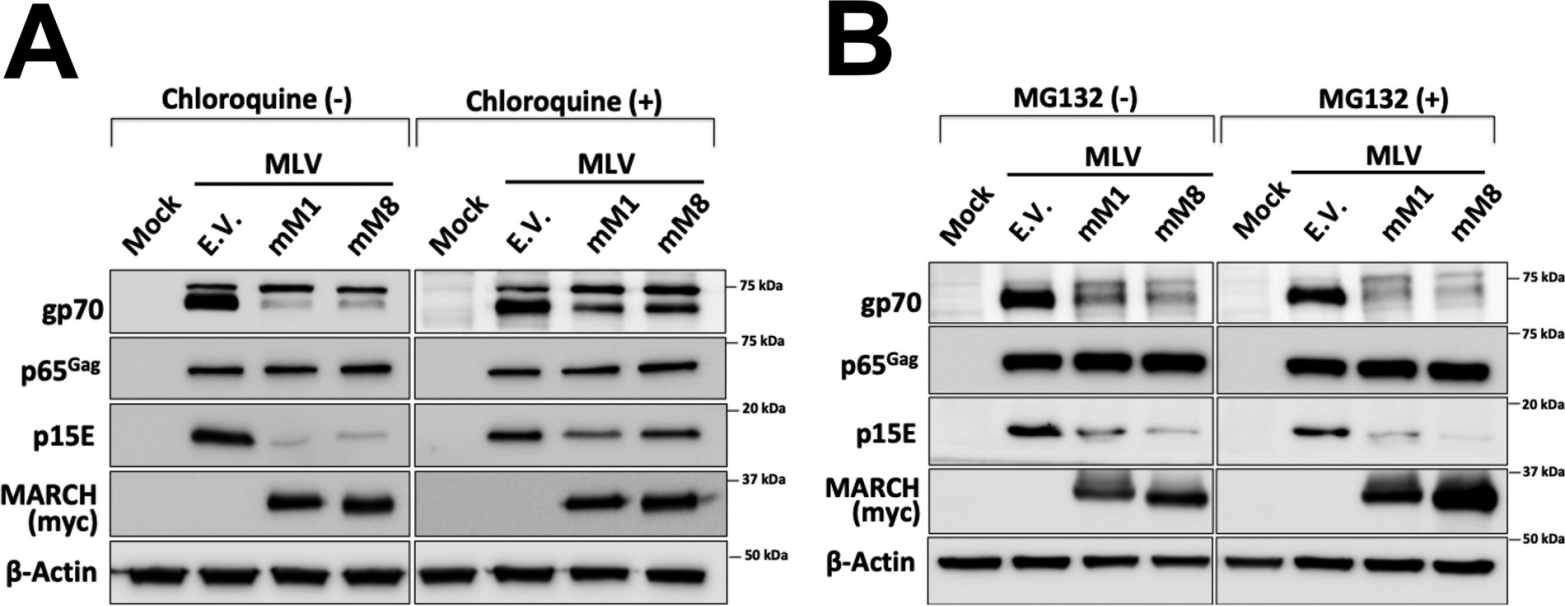
MARCH-mediated degradation of the MLV envelope glycoproteins occurs in the lysosome. (A and B) 293T cells were cotransfected with an MLV infectious clone and either mouse *March1* (*mM1*), *mM8*, or empty vector (E.V.). Cells were incubated with either (A) 100 μM chloroquine or (B) 16 μM MG132. Cells were harvested 24 h posttransfection, and lysates were analyzed by immunoblotting using anti-MLV p30 (detects p65^Gag^), anti-MLV p15E, anti-myc (for mM1 and 8 detection), and anti-β-actin antibodies. Results are shown for *n* = 3 independent experiments. Representative immunoblotting results are shown for panels A and B.

Targets of MARCH proteins are degraded in either the lysosome or the proteasome (4, 6, 23, 24). To determine where the MLV envelope glycoproteins are degraded by mouse March1 and March8, we used chloroquine and MG132, a lysosomal and a proteasomal inhibitor, respectively. 293T cells were cotransfected with either mouse *March1* or mouse *March8* along with an MLV-expressing plasmid. Six hours posttransfection, culture media were changed and cells were treated with either 100 μM chloroquine or 16 μM MG132. We observed that chloroquine treatment rescued p15E and gp70 from MARCH-mediated degradation (Fig. 7A). On the other hand, MG132 had no effect on MARCH-mediated degradation of p15E and gp70 (Fig. 7B). Therefore, we concluded that both mouse March1 and 8 result in the lysosomal degradation of MLV p15E and gp70.

### Endogenous mouse March1 and March8 restrict retrovirus infection.

**FIG 8:**
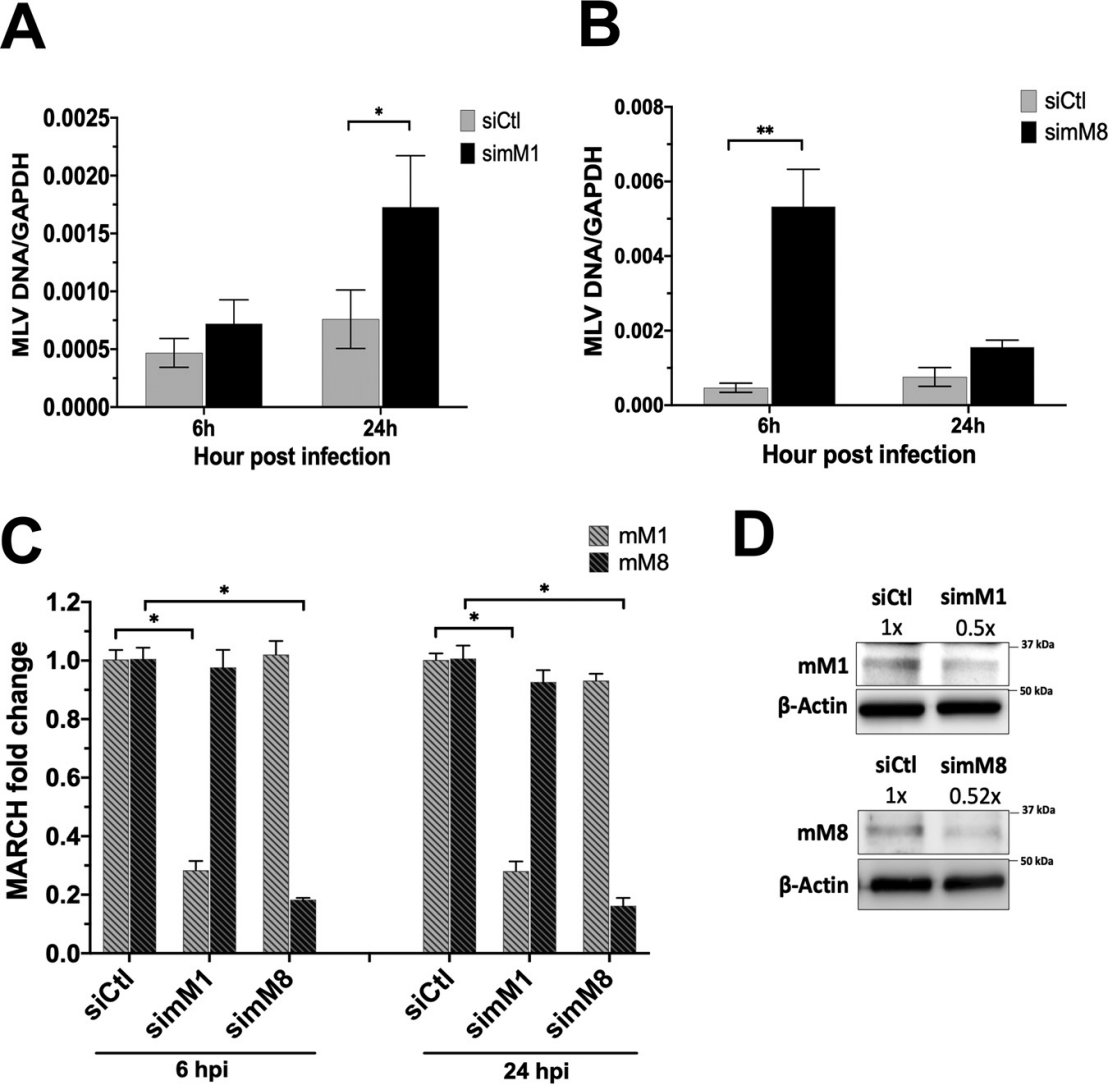
Endogenous mouse March1 and mouse March8 restrict MLV infection. (A and B) Bone marrow-derived dendritic cells (BMDCs) were transfected with the indicated siRNAs and infected with MLV (0.1 MOI). Cells were harvested 6 and 24 h postinfection, and MLV DNA levels were examined by RT-qPCR. (C and D) Knockdown verification of mouse March1 (mM1) and mM8 from siRNA-transfected BMDCs relative to siControl (siCtl) in panels A and B by either (C) RT-PCR or (D) immunoblotting. Data shown represent averages of results from *n* = 5 independent experiments. Results for panels A through C are presented as means ± standard errors of the means (SEM). Representative immunoblotting results are shown for panel D. ImageJ (NIH) was used for quantitation of the mM1 and mM8 protein levels. Statistical analysis performed using one-sample *t* test and Wilcoxon signed-rank test and unpaired *t* test. *, *P* ≤ 0.05; **, = *P* ≤ 0.01.

To determine the role of endogenous mouse March1 and 8 in retrovirus restriction, we transfected BMDCs, which express endogenous mouse March1 and 8 (Fig. 1A and B), with a mouse March1- or 8-specific small interfering RNA (siRNA) and 40 h posttransfection infected them with MLV (0.1 MOI). DNA, RNA, and protein lysates of the infected cells were analyzed by RT-qPCR (Fig. 8A, B, and C) and Western blot assays (Fig. 8D) at 6 and 24 h postinfection to measure MLV DNA levels and to ensure efficient March1 and 8 knockdown. In the case of mouse March1, we found that at 24 h postinfection the March1 knockdown-infected BMDCs had twice as much MLV DNA as those transfected with a control siRNA (Fig. 8A). For mouse March8, we noticed that at 6 h postinfection, March8 knockdown BMDCs had significantly higher levels (∼10×) of MLV DNA than BMDCs transfected with a control siRNA (Fig. 8B). We attempted to look at later time points with the infected March8 knockdown BMDCs, but we noticed significant levels of cell death, and only a small number of infected cells survived at 24 h postinfection (Fig. 8B). It is possible that the presumably high levels of MLV envelope glycoproteins in the March8 knockdown BMDCs may be toxic to the cells. Finally, we also verified that mouse March1 and 8 were efficiently knocked down (Fig. 8C and D). We concluded that while both endogenous mouse March1 and 8 restrict MLV infection, endogenous mouse March8 is more efficient in inhibiting MLV infection than mouse March1.

### MARCH proteins potently restrict a large number of disparate viral envelope glycoproteins.

**FIG 9:**
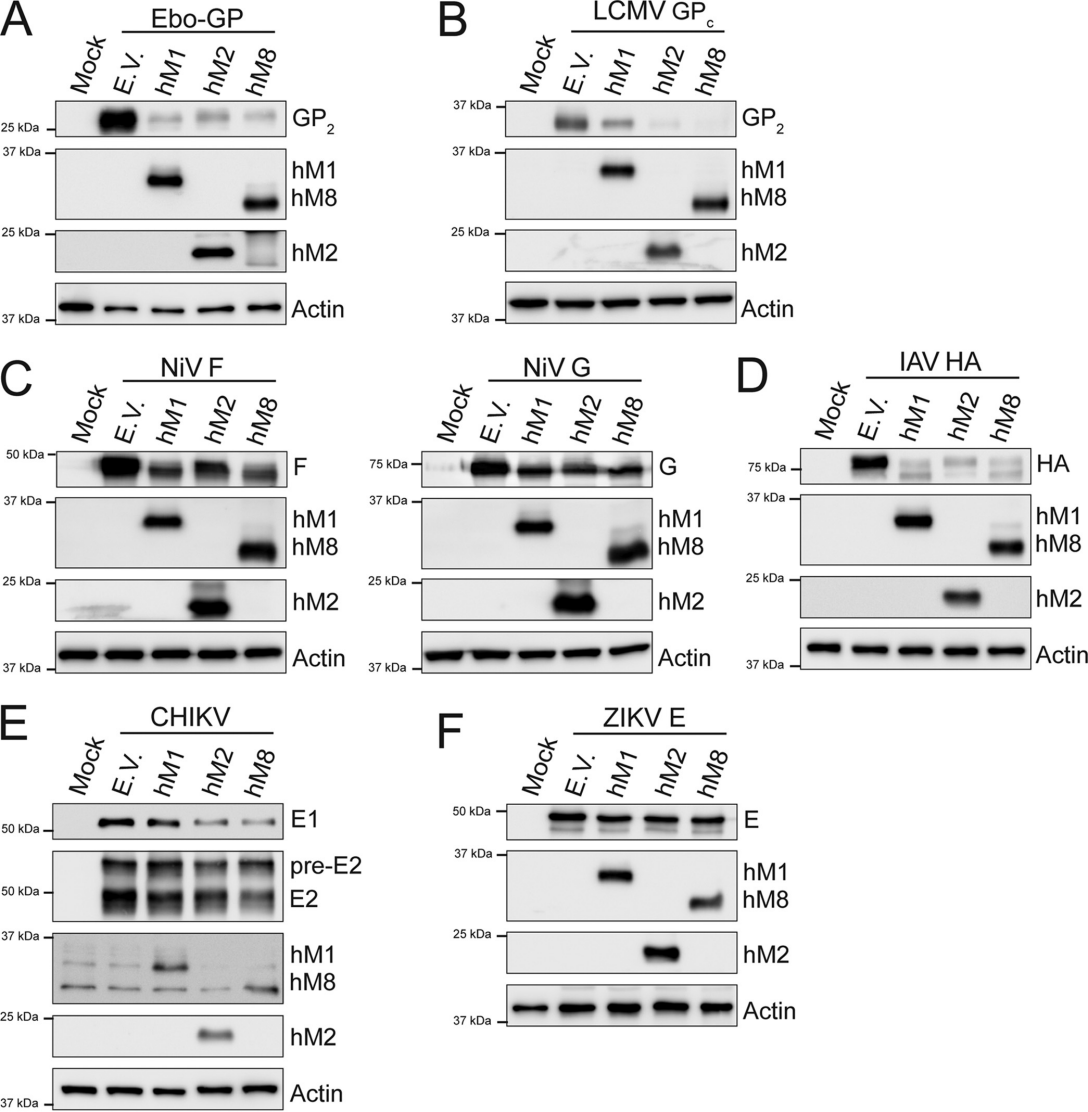
MARCH proteins target envelope glycoproteins from a diverse number of viral families. (A) Ebola virus (EBOV) mature envelope glycoprotein Gp_2_, (B) lymphocytic choriomeningitis virus (LCMV) mature envelope glycoprotein GP_2_, (C) Nipah virus (NiV) fusion glycoprotein (F) (left) and NiV receptor binding glycoprotein (G) (right), (D) influenza A virus (IAV) hemagglutinin (HA), (E) Chikungunya virus (CHIKV) E1 and E2 protein, and (F) Zika virus (ZIKV) E levels in the presence of human MARCH1 (hM1), MARCH2 (hM2), MARCH8 (hM8), and empty vector (E.V). 293T cells were cotransfected with either (A) EBOV glycoprotein (Gp), (B) LCMV Gp, (C) NiV F or NiV G, (D) IAV HA, (E) a plasmid expressing the CHIKV structural proteins, or (F) ZIKV E in combination with either *hM1*, *2*, *8*, or E.V. At 24 h posttransfection, cells were lysed and analyzed by Western blotting using anti-V5 (for EBOV GP_2_ and IAV HA detection), anti-FLAG (for LCMV GP_2_ detection), anti-AU1 (for NiV G detection), anti-HA (for NiV F detection), anti-flavivirus E antibody (4G2, for ZIKV E detection), anti-myc (CHIKV E1 detection), anti-E2 (CHIKV E2 detection), anti-MARCH1, anti-MARCH2, anti-MARCH8, and anti-β-actin antibodies. Shown are the results of a single experiment (representative of three independent experiments).

We also investigated whether MARCH proteins have a broad antiviral effect. To address this, we cotransfected 293T cells with either human *MARCH1*, *2*, or *8* and various constructs expressing a number of different viral envelope glycoproteins. Transfected cells were subsequently lysed, and Western blot assays were performed to determine viral envelope glycoprotein levels. We first examined the effect of human MARCH1, 2, and 8 on Ebola virus (EBOV), a member of the filovirus family. We observed that the levels of the mature GP_2_ subunit of the EBOV envelope glycoprotein were reduced in the presence of human MARCH1, 2, and 8 (Fig. 9A). Similar results were seen with LCMV, LASV, and Junín virus (JUNV), three members of the arenavirus family, where human MARCH1, 2, and 8 potently reduced the levels of LCMV (Fig. 9B), LASV (Fig. S4A), and JUNV GP_2_ (Fig. S4B), a subunit of the mature envelope glycoprotein complex of arenaviruses (40, 41). We also examined the effect of human MARCH1, 2, and 8 on Nipah virus (NiV), a highly pathogenic paramyxovirus. We found that human MARCH1, 2, or 8 reduced the levels of NiV fusion (F) and attachment (G) envelope glycoproteins (42) in the transfected cells (Fig. 9C). Additionally, we investigated the effect of human MARCH1, 2, and 8 on hemagglutinin (HA), a viral envelope glycoprotein of orthomyxoviruses, of the lab prototype influenza A virus (IAV) strain A/PR/8/34 (H1N1). We noticed that human MARCH1, 2, and 8 potently reduced the protein levels of IAV HA (Fig. 9D). Finally, in the case of the envelope glycoproteins E1 and E2 of Chikungunya virus (CHIKV), an alphavirus and a member of the togavirus family, only human MARCH2 and MARCH8 reduced the protein levels of both E1 and E2 (Fig. 9E).

10.1128/mBio.03264-20.4 FIG S4 Viral envelope glycoproteins vary in their susceptibility to MARCH-mediated restriction. (A) Lassa virus (LASV) mature envelope glycoprotein GP_2_, (B) Junín virus (JUNV) mature envelope glycoprotein GP_2_ (arrow), (C) human parainfluenza virus 1 (HPIV-1) hemagglutinin-neuraminidase (HN), (D) measles virus (MV) hemagglutinin (H), (E) West Nile virus (WNV) envelope (E), and (F) Crimean-Congo hemorrhagic fever virus (CCHFV) mature envelope glycoprotein G_c_ protein levels in the presence of human *MARCH1* (*hM1*), *hM2*, *hM8*, and empty vector (E.V.). 293T cells were cotransfected with either (A) LASV GPc, (B) JUNV GPc, (C) HPIV HN, (D) MV H, (E) WNV E, or (F) CCHFV M segment along with either *hM1*, *2*, *8*, or E.V. Cells were harvested 24 h posttransfection, lysed, and analyzed by Western blot assays using anti-V5 (for HPIV-1 HN and MV H detection), anti-FLAG (for LASV GP_2_ and JUNV GP_2_ detection), anti-flavivirus E antibody (clone 4G2 for WNV E detection), anti-MARCH1, anti-MARCH2, anti-MARCH8, and anti-β-actin antibodies. Shown are the results of a single experiment (representative of three independent experiments). Download FIG S4, TIF file, 0.9 MB. Copyright © 2021 Umthong et al. 2021 Umthong et al. https://creativecommons.org/licenses/by/4.0/ This content is distributed under the terms of the Creative Commons Attribution 4.0 International license.

While we observed a fairly broad antiviral effect by human MARCH1, 2, and 8 against a variety of viral envelope glycoproteins, we also identified a number of viral envelope glycoproteins that were resistant to human MARCH1-, 2-, and 8-mediated degradation. In the case of two members of the paramyxovirus family we studied, we found that both human parainfluenza virus 1 (HPIV-1) hemagglutinin-neuraminidase (HN) and measles virus (MV) hemagglutinin (H) were resistant to human MARCH1-, 2-, and 8-mediated degradation (Fig. S4C and D). Moreover, the envelope glycoproteins of Zika virus (ZIKV) and West Nile virus (WNV), two members of the flavivirus family, were also resistant to MARCH1-, 2-, and 8-mediated degradation (Fig. 9F and Fig. S4E). Finally, the Gc envelope glycoprotein of Crimean-Congo hemorrhagic fever virus (CCHFV), a member of the Orthonairovirus family, was also unaffected by human MARCH1, 2, and 8 (Fig. S4F). In summary, our findings above show that MARCH proteins can restrict viral envelope glycoproteins of a number of viruses outside the retrovirus family, further emphasizing their role as important antiviral factors.

**FIG 10:**
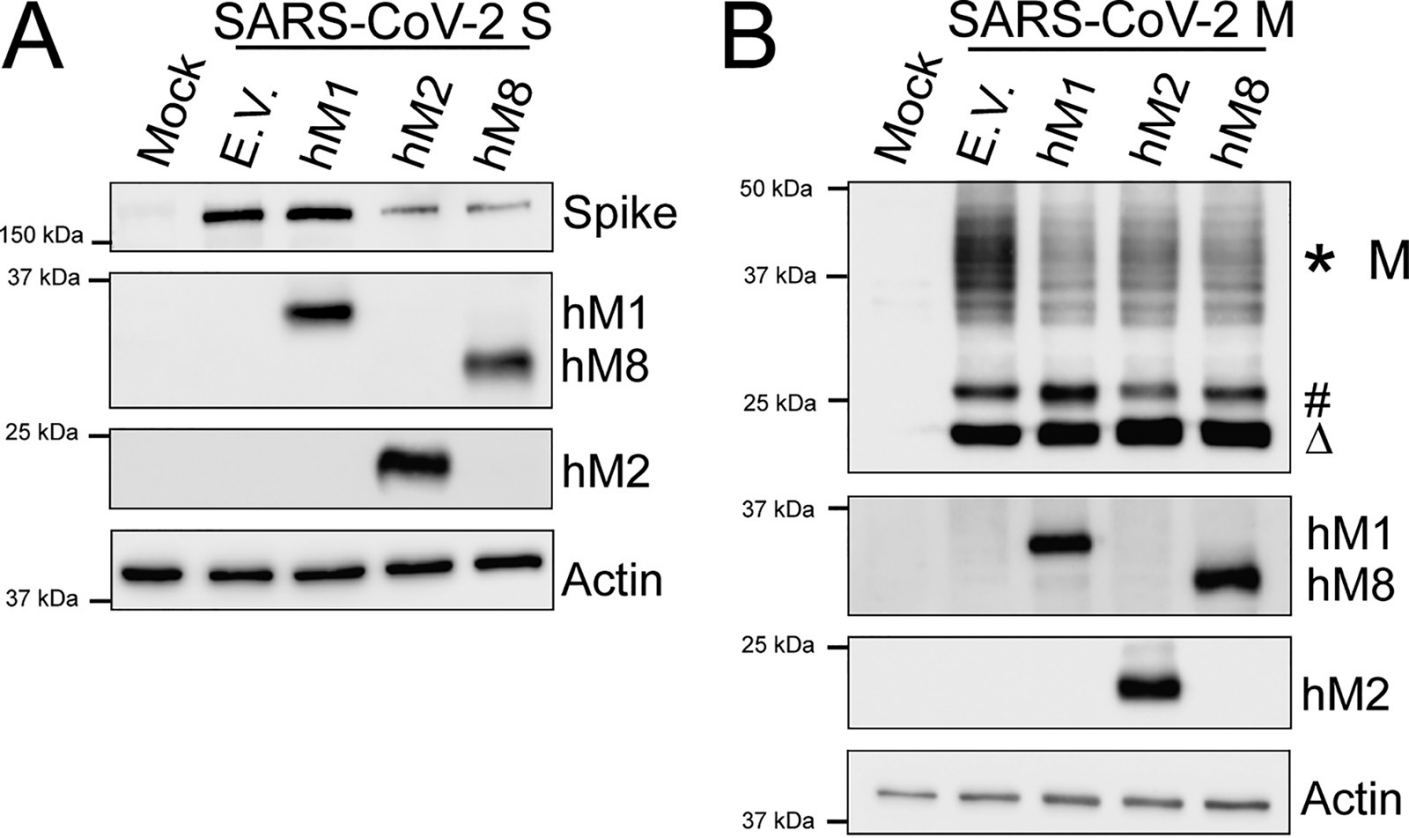
MARCH proteins potently restrict the SARS-CoV-2 Spike (S) and Membrane (M) envelope glycoproteins. (A) SARS-CoV-2 Spike (S) and (B) SARS-CoV-2 Membrane (M) cellular levels in the presence of human MARCH1 (hM1), MARCH2 (hM2), MARCH8 (hM8), and empty vector (E.V.). 293T cells were cotransfected with SARS-CoV-2 S (A) or M (B) and either *hM1*, *2*, *8*, or E.V. At 24 h posttransfection, cells were lysed and analyzed by Western blotting using anti-SARS-CoV-2 S, anti-V5 (for SARS-CoV-2 M detection), anti-MARCH1, anti-MARCH2, anti-MARCH8, and anti-β-actin antibodies. The *, #, and Δ symbols represent different glycosylated forms of SARS-CoV-2 M. Shown are the results of a single experiment (representative of three independent experiments).

A novel pathogen was recently discovered that was subsequently named severe acute respiratory syndrome coronavirus 2 (SARS-CoV-2) (43, 44). Consequently, we examined the effect of human MARCH1, 2, and 8 on the SARS-CoV-2 viral envelope glycoproteins—the S protein, which is important for receptor binding and fusion (45), and the M protein found at high levels on the viral envelope (46), which shares 90% sequence similarity with the M protein of SARS-CoV (47), is O and N glycosylated, and drives coronavirus assembly (48, 49). We found that human MARCH2 and 8 reduced S protein levels in the transfected cells, while MARCH1 had no effect (Fig. 10A). Similar to previous reports, we observed 3 forms of the SARS-CoV-2 M protein (50): a prominent diffuse band at 30 to 50 kDa, which likely represents a heterogeneous population of glycosylated SARS-CoV-2 M that is incorporated in the nascent virions and aids in immune evasion, and two distinct forms of M protein that migrate with an apparent molecular mass of 18 and 22 kDa, respectively (Fig. 10B) (50). Our data show that human MARCH1, 2, and 8 targeted specifically the highly glycosylated forms of SARS-CoV-2 M that migrate at 30 to 50 kDa, while the lower-molecular-weight forms of SARS-CoV-2 M remained unaffected (Fig. 10B). In conclusion, we show that human MARCH2 and 8 cause the degradation of two SARS-CoV-2 envelope glycoproteins, M and S, whereas MARCH1 targets only SARS-CoV-2 M for degradation.

## DISCUSSION

A number of restriction factors, including MARCH proteins, have been recently identified to target the envelope glycoproteins of retroviruses. One of these factors is guanylate-binding protein 5 (GBP5), which is a member of a family of small GTPases (51). GBP5, similarly to MARCH proteins, prevents the incorporation of the mature envelope glycoproteins in the nascent virions. However, GBP5 blocks the maturation of the retroviral envelope precursor, while MARCH proteins target the mature envelope glycoproteins at the PM (30, 32, 51). Interferon-induced transmembrane proteins (IFITMs) are another family of proteins that target the retroviral envelope glycoproteins, and similarly to MARCH proteins, they target multiple virus families including retroviruses and flaviviruses (52, 53). IFITM3 is incorporated in virions and, unlike MARCH proteins, blocks virus-cell membrane fusion (52, 54, 55). Furthermore, IFITM3, similarly to GBP5, can block the processing of the envelope glycoprotein precursor and thus decrease envelope glycoprotein incorporation in the nascent virions (56). However, the IFITM-mediated effect in retrovirus infection is not solely attributed to a reduction in envelope glycoprotein incorporation in nascent virions, as is the case with the MARCH family of proteins. In summary, the rapidly increasing number of host factors that target the retroviral envelope glycoproteins further emphasizes the importance of the retroviral envelope as the target of the host antiviral response.

We initially examined the effect of mouse IFN-β and virus infection on mouse *March1*, *2*, *3*, and *8* transcription and found that only mouse *March1* was induced by IFN-β, while MLV infection had no effect on mouse *March1*, *2*, *3*, and *8* expression. Furthermore, we found that only mouse March1 and 8 caused the degradation of the viral envelope glycoproteins of MLV and MMTV. Our findings suggest that MARCH proteins utilize a similar mechanism to target viral envelope glycoproteins and immune receptors for removal from the PM followed by degradation. Therefore, the ability of MARCH proteins to remove and degrade proteins from the cell surface is utilized by the cell in two ways: (i) for the maintenance of homeostasis, by regulating receptor levels in the PM, and (ii) for the removal and degradation of viral envelope glycoproteins as a means to thwart viral infection.

As we noticed the degradation and not the internalization and sequestration of the retroviral envelope glycoproteins, as previous studies showed, we compared side by side the effects of human and mouse MARCH1, 2, and 8 against both MLV and HIV-1 envelope glycoproteins. One of the key observations we made was that mouse March2, unlike human MARCH2, had no antiviral effect. This is particularly interesting as mouse March2 and human MARCH2 share more than 80% sequence homology (data not shown). The reason for the discrepancies in both antiviral function and transcriptional regulation between the human and mouse homologues of MARCH2 is currently unknown. Furthermore, MARCH1 also displayed a very interesting antiviral phenotype: mouse March1 was antiviral only against MLV and MMTV but not against HIV-1, whereas human MARCH1 potently downregulated envelope glycoproteins of all retroviruses tested. This suggests that human MARCH1 has acquired broader antiviral functions, allowing it to target a greater variety of retroviral envelope glycoproteins (HIV-1 and MLV). The reason behind the differences in restrictive range between mouse and human MARCH1 needs to be further elucidated. In the case of MARCH8, both mouse and human homologues degraded all retroviral envelope glycoproteins tested. To summarize, our findings suggest that MARCH proteins are important antiviral factors that are under constant selective pressure to counteract retrovirus infections.

Mouse March1 and March8 are multipass TM proteins that have both their N and C termini inside the cytoplasm (1, 15). Many domains in MARCH1 and MARCH8 have been previously characterized as essential for their ability to remove proteins from the PM. In this report, we identified that only the RING-CH and DIRT domains found in the N-terminal cytosolic tail are essential for restricting viral envelope glycoproteins. Moreover, while the C-terminal TM domain is important for mouse March1 restriction, it did not affect the March1-MLV p15E interaction. On the other hand, the C-terminal TM domain of mouse March8 is critical for restriction and interaction with MLV p15E. Our data thus suggest that mouse March1 and 8 may utilize different mechanisms to restrict MLV p15E.

We also show that the cytoplasmic tail of the MLV p15E, unlike that of HIV-1, is critical for MARCH-mediated removal from the PM, similar to cellular immune receptors targeted by MARCH proteins (6, 18, 19, 25, 57). The variability in the importance of the retroviral cytoplasmic tail in MARCH-mediated restriction further suggests that MARCH proteins may utilize distinct mechanisms to restrict envelope glycoproteins from different retroviruses.

We also examined the role of MARCH1, 2, and 8 on the viral envelope glycoproteins from a diverse number of enveloped viruses. We found that all three target the viral envelope glycoproteins of a diverse number of viruses, including NiV, EBOV, LCMV, IAV, and SARS-CoV-2. In agreement with our findings, a recent report found that EBOV Gp and IAV HA are targeted for degradation by MARCH8 (58). On the other hand, a number of viral envelope glycoproteins, including that of ZIKV and WNV, were unaffected. However, it does not necessarily mean that MARCH proteins do not restrict them; it is possible that they are sequestered intracellularly, blocking their incorporation in nascent virions, or that other members of the MARCH family are important in the degradation of these viral glycoproteins. In the case of SARS-CoV-2, we observed that human MARCH2 and 8 target both the S and M proteins while human MARCH1 targets solely the M protein for degradation. The fact that two different envelope glycoproteins of SARS-CoV-2 are restricted by a different gamut of MARCH proteins provides support to the idea that different MARCH proteins may have variable ranges of targets, something that needs to be further examined. Finally, in the case of SARS-CoV-2 M we see that MARCH proteins target only the heavily glycosylated form, which is the one that is incorporated into virions. This may be due to the fact that the heavily glycosylated form of SARS-CoV-2 M may colocalize with MARCH1, 2, and 8 at the Golgi complex (1, 9, 59) while the nonglycosylated SARS-CoV-2 M forms do not. In summary, we show that the antiviral function of MARCH proteins is well conserved among different species and is quite expansive.

## MATERIALS AND METHODS

### Cell lines.

293T cells (ATCC) and 293FT cells (Invitrogen) were cultured in Dulbecco’s modified Eagle medium (DMEM; Gibco) with 10% (vol/vol) fetal bovine serum (FBS; Sigma), 0.1 mM nonessential amino acids (Gibco), 6 mM l-glutamine (Gibco), 100 mg/ml penicillin and streptomycin (P/S; Gibco), 1 mM sodium pyruvate (Gibco), and 500 μg/ml Geneticin (Gibco). NIH 3T3 and Mus dunni cells (ATCC) were cultured in DMEM with 10% FBS and P/S. EL4 cells (ATCC) were cultured in RPMI medium with 10% FBS, P/S, and 0.05 mM β-mercaptoethanol (β-ME; Bio-Rad). MutuDC1940 cells (33) were cultured in Iscove’s modified Dulbecco’s medium (IMDM) with 8% FBS, 100 mg/ml P/S, 1 mM sodium pyruvate, 10 mM HEPES (Corning), and 0.05 mM β-ME. BMDCs and BMDMs were generated from 6- to 9-week C57BL6/N mice as previously described (60). BMDCs were differentiated with recombinant murine granulocyte-macrophage colony-stimulating factor (20 ng/ml; Invitrogen), while BMDMs were differentiated with macrophage colony-stimulating factor (10 ng/ml; Invitrogen).

### Plasmids.

The MLV molecular clone (pLRB302) and HIV-1 molecular clone (pNL4-3) used in this paper have been previously described (61, 62). The pNL4-3 was obtained from the NIH AIDS Reagent program, Division of AIDS, NIAID, NIH, from Malcolm Martin (catalog no. 114). To generate an HIV-1 infectious clone lacking the gp41 cytosolic tail (HIV-1ΔCT), we initially digested the pNL4-3 plasmid with XhoI and EcoRI. We introduced in the NL4-3 XhoI-EcoRI fragment two stop codons at residues 705 and 707 (R705Stop and R707Stop) of the envelope open reading frame (ORF) located at the N terminus of the cytosolic tail, using the Phusion site-directed mutagenesis kit (Thermo Fisher Scientific). The primers used to introduce the changes (underlined) were as follows: 5′-CTTTCTATAGTGAATTGAGTTTAGCAGGGATATTCAC-3′/5′-TACAGCAAAAACTATTCTTAAACCTACCAAGC-3′. After sequence verification, the XhoI-EcoRI fragment was reintroduced to the pNL4-3 backbone. To generate an MLV infectious clone with the cytosolic tail deleted (MLVΔCT), we introduced a stop codon in the pLRB302 plasmid at residue 640 of the envelope ORF (C640Stop) located at the N terminus of the cytosolic tail using the Phusion site-directed mutagenesis kit (Thermo Fisher Scientific) and the following primers: 5′-CTTTTTGGACCCTGAATTCTTAATCGATTAGTTC-3′/5′-CAGAATTAGTAGGAGTATAATGAGAGGCCCCAT-3′. (The change introduced is underlined in the forward primer.) The presence of the desired mutations was verified by sequencing.

Mouse *March1*, *2*, *3*, *4*, and *8* cDNA clones were purchased from Dharmacon (MMM1013-202858906, MMM1013-202761409, MMM1013-211693113, MMM1013-202799076, and MMM1013-202798072, respectively). Mouse *March1*, *2*, *3*, *4*, and *8* were initially cloned into pCDNA3.1/myc-His A (Invitrogen) and were later subcloned into pBJ5 vector using the NEBuilder HiFi DNA assembly kit (New England Biolabs). To amplify *March1*, *2*, *3*, *4*, and *8* genes from pcDNA3.1/myc-His A, the same reverse primer was used for all mouse *March* genes, 5′-GGCCTCCGCGGCCGCTCAATGGTGATGGTGATGAT-3′, while the forward primers were different for all genes and are the following: for *mM1*, 5′-GCTCTAGCCCTCGAGATGCCCCTCCACCAGATTTC-3′; for *mM2*, 5′-GCTCTAGCCCTCGAGATGACGACAGGTGACTGTTGCC-3′; for *mM3*, 5′-GCTCTAGCCCTCGAGATGACAACCAGTCGCTGCAGTC-3′; for *mM4*, 5′-GCTCTAGCCCTCGAGATGCTCATGCCCCTGGGTGG-3′; for *mM8*, 5′-GCTCTAGCCCTCGAGATGAGCATGCCATTGCACCAG-3′. To amplify the pBJ5 vector, the following forward primer was used for all mouse *March* genes: 5′-ATCACCATTGAGCGGCCGCGGAGGCCGAATTC-3′. The reverse primers used to amplify pBJ5 differed among the different mouse *March* genes and are the following: for *mM1*, 5′-TGGTGGAGGGGCATCTCGAGGGCTAGAGCAGCTTTTAGAG-3′; for *mM2*, 5′-GGCAACAGTCACCTGTCGTCATCTCGAGGGCTAGAGCAGCTTTTAGAG-3′; for *mM3*, 5′-GACTGCAGCGACTGGTTGTCATCTCGAGGGCTAGAGCAGCTTTTAGAG-3′; for *mM4*, 5′-CCACCCAGGGGCATGAGCATCTCGAGGGCTAGAGCAGCTTTTAGAG-3′; for *mM8*, 5′-CTGGTGCAATGGCATGCTCATCTCGAGGGCTAGAGCAGCTTTTAGAG-3′. PCR products were amplified and recombined using the NEBuilder HiFi DNA assembly kit (New England Biolabs) followed by Sanger sequencing for identification of positive clones. In the case of human *MARCH* genes, hM1 cDNA was synthesized from HeLa cell RNA. The pCMV-hM2-3HA has been previously described (31) and kindly provided to us by Xinqi Liu, while cDNA of hM8 was obtained from Dharmacon (MHS6278-202807570). All human *MARCH* cDNAs were cloned into pCDNA3.1/myc-His A (Invitrogen) and were then subcloned into pBJ5. hM1, 2, and 8 were initially PCR amplified using a common reverse primer, 5′-GGCCTCCGCGGCCGCTCAATGGTGATGGTGATGAT-3′, and the following forward primers: for hM1, 5′-GGCTCGAGATGCTGGGCTGGTGTGAAGCG-3′; for hM2, 5′-GGCTCGAGATGACGACGGGTGAC-3′; and for hM8, 5′-GGCTCGAGATGAGCATGCCACTG-3′. The PCR fragments and pBJ5 were cut with NotI and XhoI followed by ligation, transformation, and sequencing for positive clones. To generate all the mM1 and mM8 mutants described in this paper, we used the Phusion site-directed mutagenesis kit (Thermo Fisher Scientific). To generate the RING-CH^mut^ mM1 and mM8, mutations were introduced at C80S, C83S, C97S, and C99S using the primers described in Table S1 in the supplemental material. To generate mM1 and mM8 ΔDIRT, we deleted residues 130 to 140 using the primers described in Table S1. To mutate the tyrosine endocytic motifs (YXXΦ) of mM1 (^218^YVQL^221^ and ^228^YNRL^231^) and mM8 (^218^YLQL^221^ and ^228^YNRL^231^), we mutated them to ^218^AAQL^221^ and ^228^AARL^231^ for both mM1 and mM8 using the primers shown in Table S1. The ^234^VQNC^237^ motifs of mM1 and mM8 were mutated to ^234^AANC^237^ using the primers mentioned in Table S1. To generate mM1 and mM8 carrying the TM domains of mM3 and mM4, respectively, we initially PCR amplified the TM domains of mM3 and mM4 with the primers shown in Table S1. We then PCR amplified mM1 and mM8 with primers flanking their TM domains and described in Table S1. PCR products were recombined using the NEBuilder HiFi DNA assembly kit (New England Biolabs). All constructs were verified by sequencing.

10.1128/mBio.03264-20.5 TABLE S1 Primers for making mM1 and mM8 mutants. Download Table S1, DOCX file, 0.01 MB. Copyright © 2021 Umthong et al. 2021 Umthong et al. https://creativecommons.org/licenses/by/4.0/ This content is distributed under the terms of the Creative Commons Attribution 4.0 International license.

The influenza A virus (IAV) hemagglutinin (HA) gene was PCR amplified from cDNA generated from IAV A/Puerto Rico/8/1934 (H1N1) genomic RNA (BEI Resources, NIAID, NIH, NR-2773) using the primers described in Table S2 followed by restriction digestion with BamHI and XbaI and ligation into pcDNA-V5/His (Invitrogen). The MV H and HPIV-1 HN were PCR amplified using the primers shown in Table S2 from genomic RNA of MV, Edmonston strain, and from virus of the HPIA1-HN-F/HPIA1-HN-R strain of HPIV-1 (BEI Resources, NIAID, NIH, NR-44104 and NR-48681, respectively) and cloned into pcDNA-V5/His (Invitrogen). The M fragment of CCHFV was PCR amplified with primers shown in Table S2 from cDNA generated from CCHFV genomic RNA, IbAr10200 strain (BEI Resources, NIAID, NIH NR37382). PCR products were cloned into pcDNA-V5/His (Invitrogen) using the NEBuilder HiFi DNA assembly master mix (New England Biolabs). SARS-CoV-2 M was PCR amplified from cDNA derived from genomic RNA of SARS-CoV-2, isolate USA-WA1/2020 (BEI Resources, NIAID, NIH, NR-52285), using the primers in Table S2 and cloned into pCDNA-V5/His TOPO (Invitrogen). We introduced a FLAG tag to JUNV GP by PCR amplifying it from a previously described expression plasmid (63, 64) using the primers in Table S2, and after digestion with BamHI and XhoI, it was cloned into pCDNA^TM^3.1/myc-His A (Invitrogen). CHIKV structural genes were initially PCR amplified from pDONR21-CHKVstr (65) kindly provided to us by Ted Pierson, using the primers shown in Table S2, and cloned into pcDNA-V5/His (Invitrogen). pCAGGS-EboGP-V5 (strain Zaire 1976 Mayinga) (66) and pcDNA V5/His SARS-CoV-2 Spike (Wuhan-Hu-1) were kindly provided by Paul Bates. LASV and LCMV GPs tagged with a FLAG tag have been previously described (67, 68). pcDNA 3.1(+) ZIKA ENV was provided by Amy Jacobs. Plasmids encoding codon-optimized NiV F and NiV G genes tagged with AU1 and HA tag, respectively, were kindly provided by Benhur Lee (69). pVAC2-WNV prME plasmid was kindly given to us by Ted Pierson (70).

10.1128/mBio.03264-20.6 TABLE S2 Primers for cloning of viral envelopes. Download Table S2, DOCX file, 0.01 MB. Copyright © 2021 Umthong et al. 2021 Umthong et al. https://creativecommons.org/licenses/by/4.0/ This content is distributed under the terms of the Creative Commons Attribution 4.0 International license.

### Transfections and Western blots.

For all transfection experiments, we used Lipofectamine 3000 transfection reagent (Thermo Fisher Scientific) according to the manufacturer’s recommendation. 293T cells were seeded on a 6-well plate (0.5 × 10^6^ cells/well) and the following day were cotransfected with 3 μg of MLV (pLRB3020) or 3 μg of pNL4.3 along with either 4 μg of pBJ5-*mMarchs* (*mM1*, *mM2*, *mM3*, or *mM8*) or 4 μg of pBJ5-*hMARCHs* (*hM1*, *hM2*, or *hM8*). To study the dose-dependent effect of *mM1* and *mM8*, we used the following concentrations of *mM1* and *mM8*: 0.25, 0.5, 1, 2, and 4 μg. For the MMTV transfection experiments, we seeded 293T cells in a 6-well plate (0.5 × 10^6^/well) and transfected them with 5 μg of MMTV hybrid provirus (HP) plasmid (34), 50 ng of rat glycocorticoid receptor (RSVGR) construct, and 1 μg *mM1*, *2*, *3*, or *8* or E.V. (1 μg). For the cotransfection experiments with the mutant *mM1* and *mM8* constructs, we seeded 0.5 × 10^6^ 293T cells, and the following day we cotransfected them with 3 μg of MLV infectious clone (pLRB302) and 1 μg of either E.V., *mM1*, *mM8*, or *mM1* and *mM8* with mutations in the various domains mentioned under “Plasmids.” To determine the role of the cytosolic tail of MLV p15E and HIV-1 gp41, we cotransfected 1 μg of *mM1* or *mM8* with 3 μg of either wild-type MLV, wild-type HIV-1, MLVΔCT, or HIV-1ΔCT. For all above transfections, cells and culture media were harvested 48 h after transfection. Cells were lysed in RIPA buffer (150 mM NaCl, 1% NP-40, 0.5% sodium deoxycholate, 0.1% SDS, 25 mM Tris, pH 7.4, with Halt phosphatase and protease inhibitors), and viruses from the culture media were used for future experiments. Cell lysates and virus pellets were mixed with 1× sample loading buffer before they were resolved on 10% or 15% sodium dodecyl sulfate-polyacrylamide gels. Blots were probed using the following antibodies: goat anti-MLV gp70 (71), goat anti-MMTV polyclonal (72), rat anti-MLV transmembrane protein/p15E (clone 42/114; Kerafast), rabbit anti-myc (Cell Signaling Technology), rat anti-MLV p30 (R187, ATCC CRL-1912), mouse anti-HIV-1 envelope (16H3; NIH/AIDS Reagent program catalog no. 12559), mouse anti-HIV gp41 (Chessie8) (NIH/AIDS Reagent program catalog no. 526), human anti-HIV gp41 clone 2F5 (NIH/AIDS Reagent program catalog no. 1475) for detection of gp41ΔCT, mouse anti-HIV p24 (NIH/AIDS Reagent program catalog no. 4121), rabbit anti-MARCH1 (Thermo Fisher Scientific catalog no. PA5-20632), rabbit anti-MARCH2 (Thermo Fisher Scientific catalog no. PA5-30220), rabbit anti-MARCH8 (Thermo Fisher Scientific; PA5-20632), and monoclonal anti-β-actin (Sigma-Aldrich). Horseradish peroxidase (HRP)-conjugated anti-rabbit IgG (Cell Signaling Technology), HRP-conjugated anti-rat IgG (Cell Signaling Technology), HRP-conjugated anti-human IgG (Sigma-Aldrich, catalog no. GENA933), HRP-conjugated anti-mouse (EMD Millipore), and HRP-conjugated anti-goat (Sigma-Aldrich) were used for detection using the enhanced chemiluminescence detection kits Clarity and Clarity Max ECL (Bio-Rad).

To determine the effect of MARCH proteins on the nonretroviral envelope glycoproteins, we seeded 8 × 10^5^ 293T cells in a 6-well plate and cotransfected them with 4 μg of either *hM1*, *2*, *8*, or E.V. and 50 ng of EBOV GP-V5, LCMV GP-FLAG, ZIKV E, LASV GP-FLAG, SARS-CoV-2 M-V5, or SARS-CoV-2 S. In the case of IAV HA, 4 μg of *hM1*, *2*, *8*, or E.V. was cotransfected with 100 ng of IAV HA-V5. For NiV F and G, 25 ng of either NiV F-AU1 or NiV G-HA was cotransfected with 4 μg of either *hM1*, *2*, *8*, or E.V. For CCHFV Gc, CHIKV E1 and E2, MV H, and HPIV-1 HN, we cotransfected 4 μg of the viral envelopes along with 700 ng of *hM1*, *2*, *8*, or E.V. In the case of WNV, we cotransfected 400 ng of WNV PrmE along with 1 μg of *hM1*, *2*, *8*, or E.V. For JUNV, we cotransfected 5 μg of JUNV Gp-FLAG and 1 μg of *hM1*, *2*, *3*, *8*, or E.V. Cells were harvested 48 h posttransfection and lysed in RIPA buffer except for MV H and HPIV HN, which were lysed in DM lysis buffer (0.5% [wt/vol] *n*-decyl-β-d-maltopyranoside, 20 mM Tris-HCl, pH 7.5, 10% [vol/vol] glycerol, 1× Halt protease inhibitor cocktail [Thermo Fisher Scientific], Benzonase [25 U/ml]). Lysates were then resolved on 10% sodium dodecyl sulfate-polyacrylamide gels, and blots were probed using the following antibodies: rabbit anti-FLAG (Cell Signaling Technology), rabbit anti-V5 (Invitrogen), mouse anti-flavivirus envelope 4G2 (BEI Resources, NIH, NIAID NR-50327), rabbit anti-AU1 (Novus Biologicals), mouse anti-CHIKV E2 (BEI Resources, NIH, NIAID NR-44002), mouse anti-SARS-CoV-2 S (GeneTex catalog no. 632604), and the rabbit anti-hM1, 2, and 8 described above. HRP-conjugated anti-rabbit and anti-mouse were used for detection using the chemiluminescence detection kit Clarity ECL (Bio-Rad).

### FACS analysis.

The purity of the BMDM and BMDC populations used in our experiments was determined by cell surface staining followed by fluorescence-activated cell sorting (FACS). For BMDMs, we stained 1 × 10^5^ BMDMs with 1:50 of fluorescein isothiocyanate (FITC) rat anti-mouse CD11b (clone M1/70; BDPharmingen catalog no. 557396) in PBS with 2% FBS (staining buffer) for 30 min at 4°C. Cells were washed with 500 μl of staining buffer twice, resuspended in 100 μl of staining buffer, and processed using a BD LSRFortessa flow cytometer. For BMDCs, 1 × 10^5^ BMDCs were harvested and stained with a 1:25 dilution of allophycocyanin (APC) hamster anti-mouse CD11c (clone HL3; BDPharmingen catalog no. 550261) in staining buffer, washed, and run through a BD LSRFortessa flow cytometer.

To verify that the March1 mutants we generated localized to the plasma membrane, we seeded 0.5 × 10^6^ 293T cells, and the following day, cells were transfected with 3 μg of E.V., *mM1*, or the mutant forms of *mM1* using Lipofectamine 3000 (Thermo Fisher Scientific) per the manufacturer’s recommendation. Cells were harvested, and 1 × 10^5^ cells were stained with 1:50 of rabbit anti-MARCH1 (Thermo Fisher Scientific catalog no. PA5-69223) or 1:50 of rabbit IgG isotype antibody (Thermo Fisher Scientific catalog no. 02-6102) for 30 min at 4°C. Subsequently, cells were washed twice and stained with 1:50 of goat anti-rabbit IgG (H+L) Alexa Fluor 647 (Thermo Fisher Scientific catalog no. A21244) for 30 min at 4°C followed by two washes and dilution in 100 μl of staining buffer. Samples were processed using a BD LSRFortessa flow cytometer. Stained populations from our FACS experiments were further analyzed using FlowJo software version 10.7.1.

### Membrane fractionation of mM8.

293T cells at 5 × 10^5^ were seeded and the next day transfected using Lipofectamine 3000 (Thermo Fisher Scientific) with 3 μg of E.V., wild-type mM8, or the mutant mM8 constructs we generated. At 24 h posttransfection, cells were collected and membranes were extracted using the Mem-PER Plus membrane extraction kit (Thermo Fisher Scientific) according to the manufacturer’s guidelines. The purity of the membrane fractions was verified by Western blot assays probing with rabbit anti-GAPDH (Cell Signaling Technology).

### Virus infection of BMDCs.

BMDCs were seeded (5 × 10^4^ cells/well) in a 96-well plate. The following day, cells were transfected with 3 pmol of siRNA control (Ambion catalog no. AM4611), simM1 (Ambion ID: s91382; sense, CGUGUGAUCUUUGUGCAGATT; antisense, UCUGCACAAAGAUCACACGGT), or simM8 (Ambion ID: s90045; sense, ACUCAAGGCUUACAAUAGATT; antisense, UCUAUUGUAAGCCUUGAGUCT) using Lipofectamine RNAiMax (Thermo Fisher Scientific) according to the manufacturer’s recommendation. At 40 h posttransfection, cells were infected with an 0.1 MOI of MLV by spinoculation as previously described (73). Cells were harvested, and DNA was isolated using the DNeasy blood and tissue kit (Qiagen) at the indicated time points according to the manufacturer's instructions. RT-qPCR was performed using the Power Up SYBR Green master mix kit (Applied Biosystems) and the previously described MLV and GAPDH primers (74). A CFX384 Touch real-time PCR detection system (Bio-Rad) was used for all RT-qPCR assays described in this study.

### siRNA knockdown verification.

BMDCs were transfected with the indicated siRNAs as described above, and RNA was isolated using the RNeasy minikit (Qiagen). cDNA was generated using the SuperScript III first-strand cDNA synthesis kit (Invitrogen). RT-PCR was performed using the Power Up SYBR Green master mix kit (Applied Biosystems) and the following primers: mM1, 5′-TCTGCTCTGTCACGTTCCAC-3′/5′-CCTCTGCAGTTGGCAGTGTA-3′; mM8, 5′-CTCTCGCACTTCTATCACGCCA-3′/5′-AAGTGGAGGCTTCCTGTGCAGT-3′; and GAPDH (described under “Virus infection of BMDCs”). RT-PCRs were performed as described above.

To detect the knockdown efficacies at the protein level, cells were lysed with RIPA buffer. Cell lysates were resolved on 10% sodium dodecyl sulfate-polyacrylamide gels. Blots were probed with the following antibodies: rabbit anti-MARCH1 (Invitrogen catalog no. PA5-69223), rabbit anti-MARCH8 (Invitrogen catalog no. 30220), and monoclonal anti-actin (Sigma-Aldrich). HRP-conjugated anti-mouse (EMD Millipore) and HRP-conjugated anti-rabbit (Cell Signaling Technology) were used for detection using the chemiluminescence detection kits Clarity and Clarity ECL (Bio-Rad).

### Interferon treatment of cells.

MutuDC1940, EL-4, and NIH 3T3 cells, BMDMs, and BMDCs (all at 0.5 × 10^4^) were seeded in a 96-well plate for 24 h. Cells were then treated ±500 units/ml of mouse IFN-β (PBL Assay Science) for 4, 8, 16, and 24 h. For the titration experiment, 0.5 × 10^4^ MutuDC1940 cells were treated with IFN-β concentrations ranging from 0.005 to 5,000 units/ml and harvested 4 h posttreatment. Cells were lysed, and RNA was isolated using an RNeasy minikit (Qiagen). cDNA synthesis and RT-PCR were performed as described under “siRNA knockdown verification” using the aforementioned mM1, mM8, and GAPDH primers as well as primers for mM2 (5′-TGCCAGCTGTACTCGGAATG-3′/5′-GCTGCATTGCCATCTGACTC-3′) and for mM3 (5′-ATCAGTCGAGCAGAAGCTGAG-3′/5′-AGTGTCAGCCTCGTCACATC-3′).

### Virus preparation.

MLV stocks were prepared by transfecting 293FT cells (Invitrogen) seeded in 10-cm-diameter cell culture dishes using Lipofectamine3000 (Thermo Fisher Scientific) with 25 μg of an MLV infectious clone (pLRB302) per the manufacturer’s recommendation. Culture supernatants were harvested 48 h after transfection, filtered, and treated with 10 U/ml DNase I (Roche) for 40 min at 37°C. Titers of viruses were determined in Mus dunni cells as previously described (74).

### Infection assays.

To examine the effect of MLV infection on MARCH gene expression, 0.5 × 10^4^ cells of MutuDC1940, EL4, and NIH 3T3 were seeded in a 96-well plate followed by infection with MLV (5 MOI) via spinoculation as previously described (73). Following spinoculation, cells were given fresh medium and harvested at 4, 8, 16, and 24 h post infection followed by RNA isolation using an RNeasy minikit (Qiagen) per the manufacturer’s recommendations. Primers for mM1, 2, 3, 8, and GAPDH detection are mentioned above.

### Coimmunoprecipitations.

For our coimmunoprecipitation experiments examining endogenous mM1 and 8, we seeded 2 × 10^5^ MutuDC1490 cells in a 6-well plate. The next day, cells were either mock infected (medium) or infected by spinoculation with MLV (10 MOI) as mentioned above. Three days postinfection, cells were washed once with cold 1× PBS and harvested using NP-40 lysis buffer (50 mM Tris-HCl, 150 mM NaCl, 1% NP-40, 5 mM EDTA, 5% glycerol). Coimmunoprecipitation was performed using the Dynabeads protein A immunoprecipitation kit (Thermo Fisher Scientific) following the manufacturer’s protocol with some modifications. Briefly, 50 μl protein A Dynabeads (Thermo Fisher Scientific) was preincubated with 2 μg (1:50 dilution) of rabbit anti-MARCH1 (Invitrogen catalog no. PA5-69223) or with 0.5 μg (1:25 dilution) of rabbit anti-MARCH8 (Proteintech catalog no. 14119-1-AP) or with an equal amount of rabbit IgG isotype antibody (Thermo Fisher Scientific catalog no. 02-6102) antibodies. Cell lysates were incubated with antibody-coated protein A Dynabeads overnight at 4°C, washed, and eluted. The eluted fractions were subjected to SDS-PAGE and Western blot analysis. For our coimmunoprecipitation experiments examining the role of the mM1 and 8 TM domains, we cotransfected 293T cells with 3 μg of an MLV infectious clone (pLRB302) and 100 ng of the indicated different mouse *March* constructs. At 24 h posttransfection, cells were lysed in NP-40 lysis buffer. Protein A Dynabeads were preincubated with anti-Myc (Cell Signaling Technology) or 1:20 of culture supernatant of 372 (ATCC CRL-1893), and then cell lysates were added to antibody-bound protein A Dynabeads, incubated at room temperature for 1 h, washed, and eluted, followed by SDS-PAGE and Western blot analysis detecting MLV p15E or March (myc).

### Luciferase assays.

293T cells were seeded in a 6-well plate (0.5 × 10^6^ cells/well) a day prior to transfections. The following day, cotransfection was performed using 3 μg of an MLV infectious clone (pLRB302), 1 μg of pFB-Luciferase (pFB-*luc*) plasmid, and 4 μg of either pBJ empty vector, pBJ5-*mM1*, pBJ5-*mM2*, pBJ5-*mM3*, or pBJ5-*mM8*, using Lipofectamine 3000 (Thermo Fisher Scientific) per manufacturer’s recommendation. Medium was changed 24 h posttransfection, and 24 h later, culture supernatants were collected, spun down at 3,000 rpm for 10 min, filtered, and stored at −80°C. NIH 3T3 cells (0.2 × 10^6^ cells/well) were seeded in a 6-well plate and the next day were infected with luciferase reporter viruses normalized for equal amounts of p30 by Western blot analyses. Medium was changed 24 h postinfection, and luciferase levels were measured 48 h postinfection using the Steady-Glo luciferase assay system (Promega) per the manufacturer’s recommendation and an automated plate reader, the Biostack4 (BioTek) luminometer.

### Chloroquine and MG132 treatment.

293T cells at 5 × 10^5^/well were seeded in a 6-well plate. Cells were cotransfected with 3 μg of an MLV infectious clone along with either 1 μg of E.V., *mM1*, or *mM8*. At 6 h posttransfection, fresh medium ± 100 μM chloroquine (Sigma-Aldrich) or ± 16 μM MG132 (Sigma-Aldrich) was added. Cells were harvested and lysed in RIPA buffer 18 h later followed by SDS-PAGE and Western blot analysis.

### Statistical analysis.

Statistical analyses were performed using GraphPad Prism software version 8.2. The statistical tests used to determine significance are described in the figure legends. A difference was considered to be significant for *P* values of <0.05.
