# Supplementary figures and images for: Elucidating the Antiviral Mechanism of Different MARCH Factors
Source: mBio. 2021 Mar 2;12(2):e03264-20. doi: 10.1128/mBio.03264-20 (PMC8092282; doi:10.1128/mBio.03264-20)

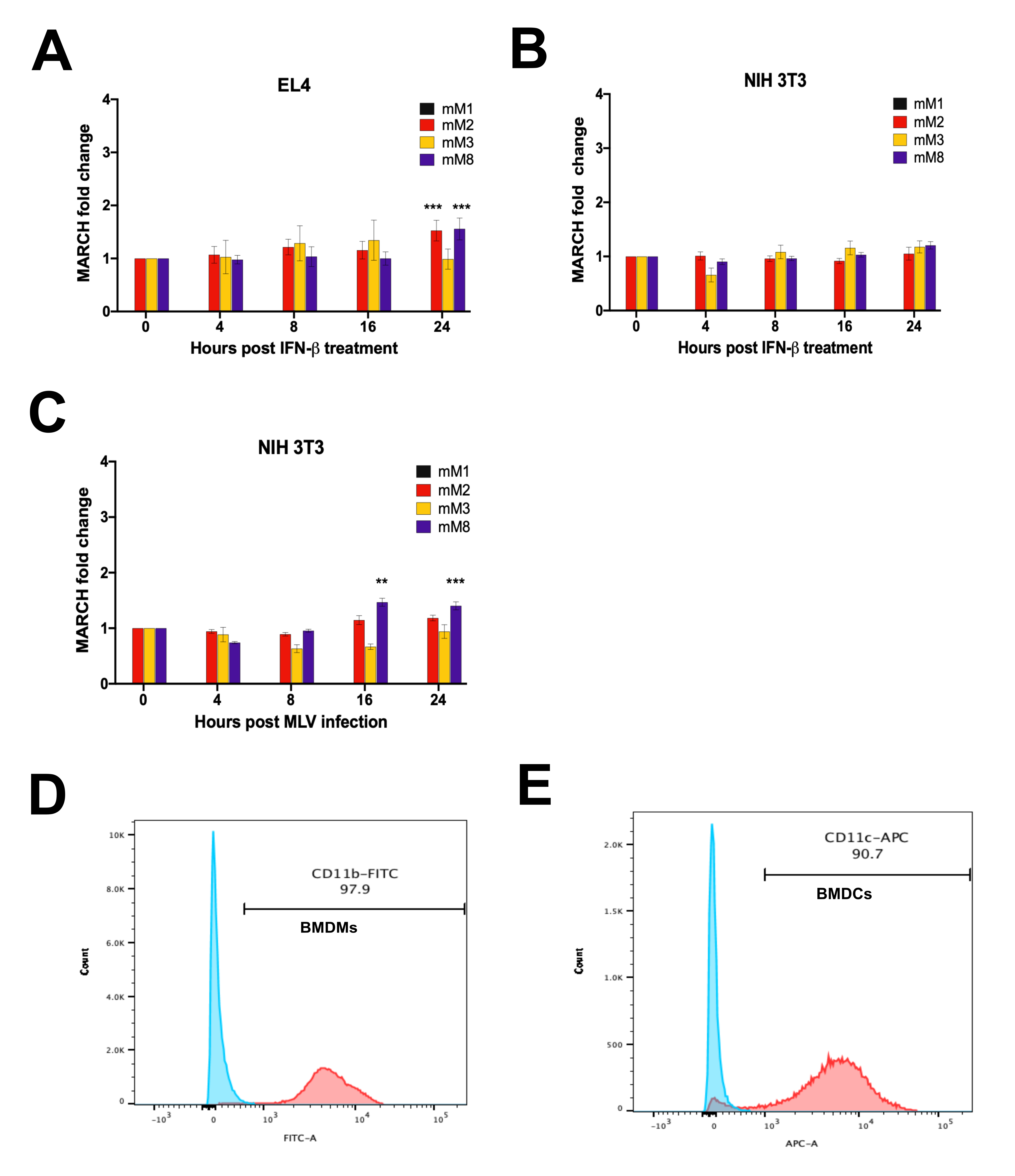

Supplement: FIG S1 [file mBio.03264-20-sf001.tif]

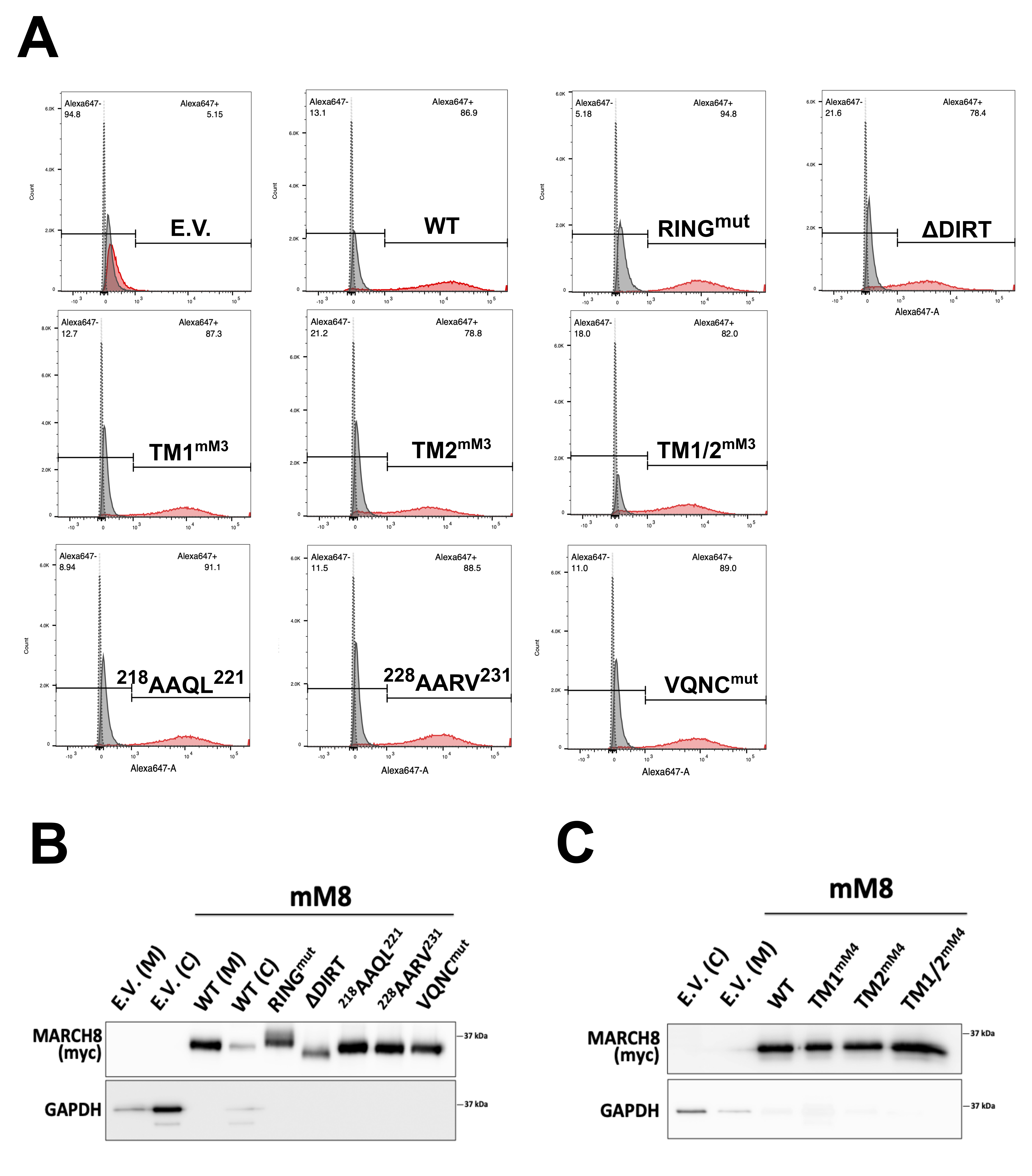

Supplement: FIG S2 [file mBio.03264-20-sf002.tif]

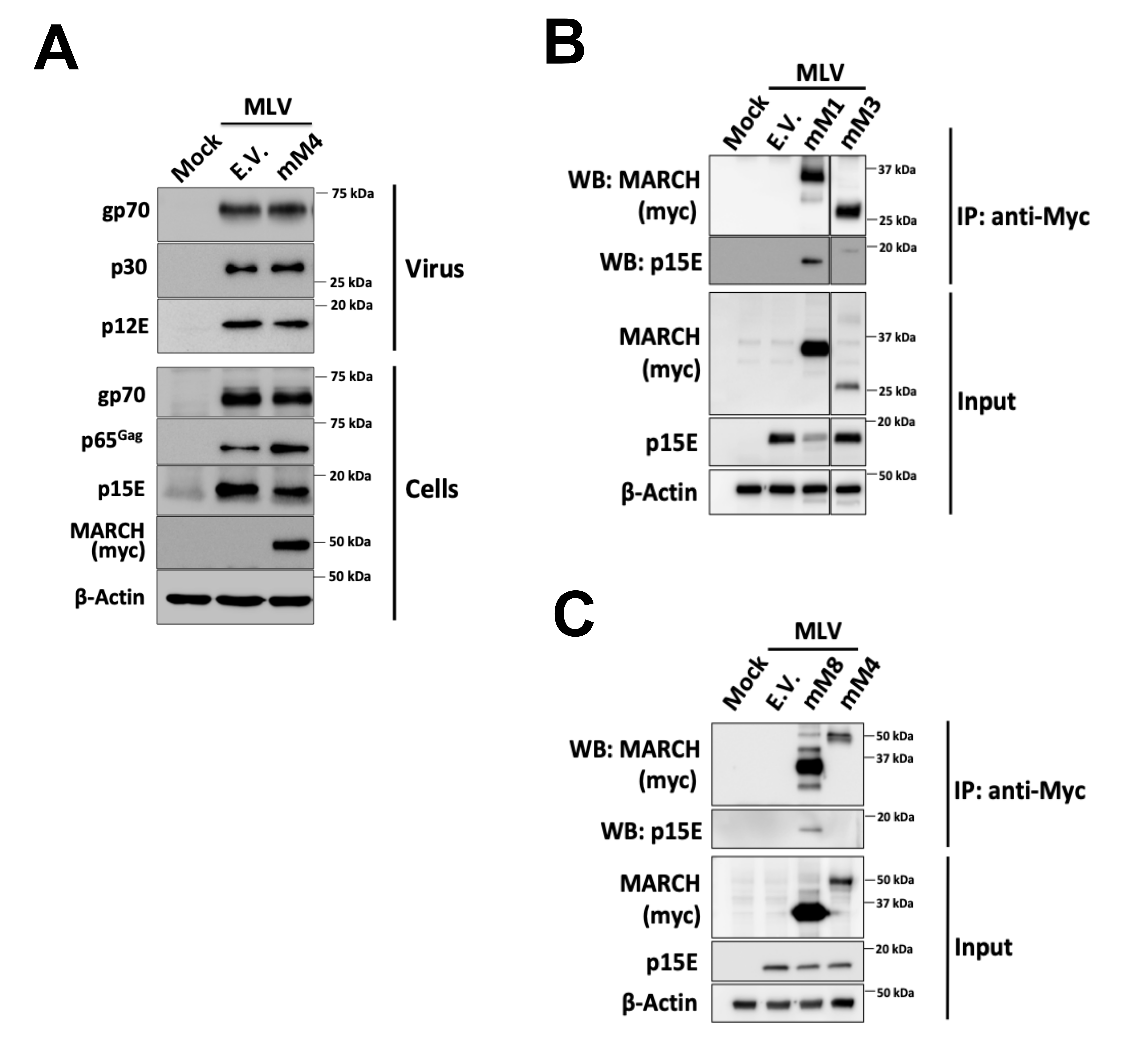

Supplement: FIG S3 [file mBio.03264-20-sf003.tif]

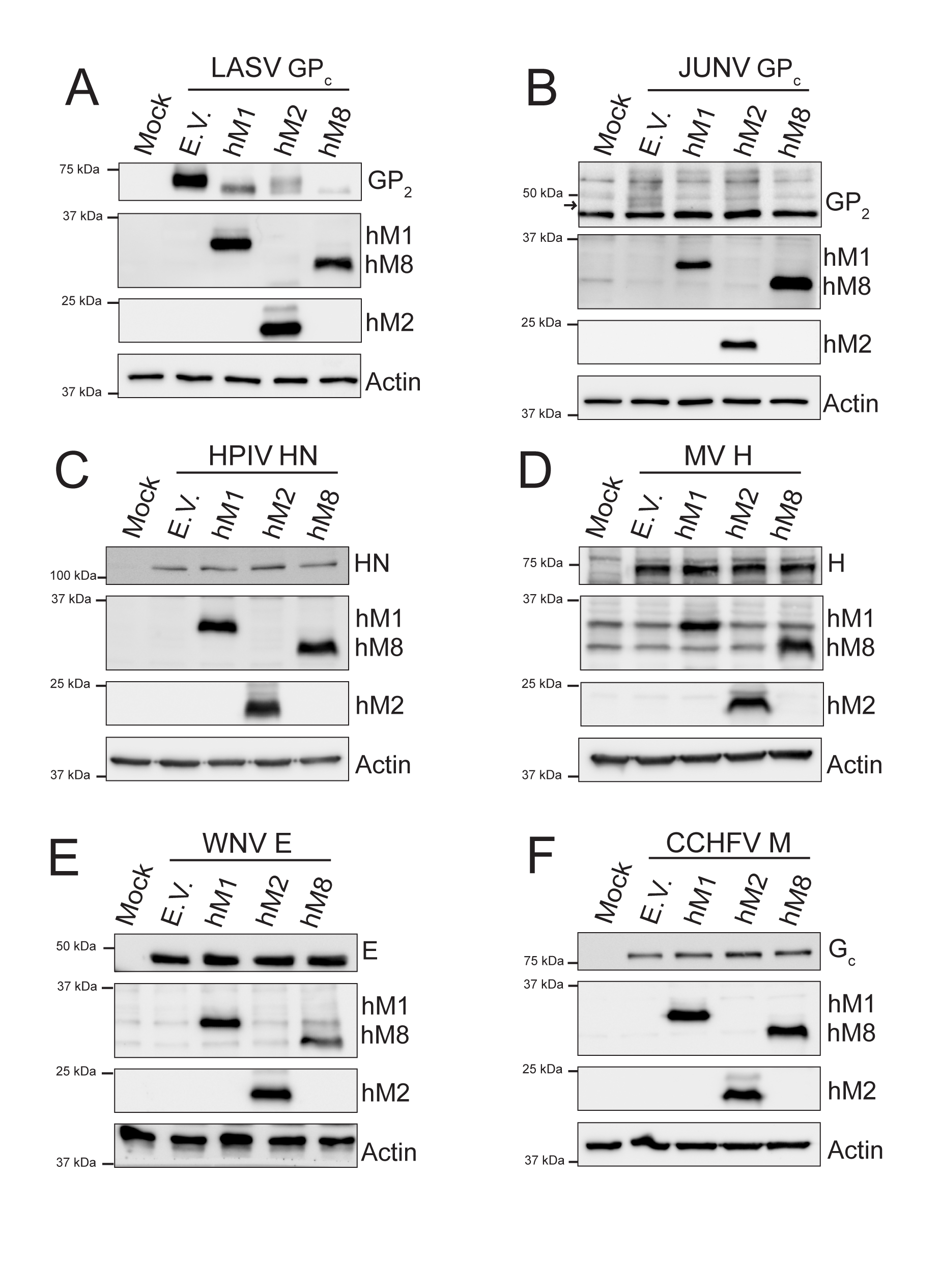

Supplement: FIG S4 [file mBio.03264-20-sf004.tif]
