# Supplementary material for: Elucidating the Antiviral Mechanism of Different MARCH Factors
Source: mBio. 2021 Mar 2;12(2):e03264-20. doi: 10.1128/mBio.03264-20 (PMC8092282; doi:10.1128/mBio.03264-20)
Supplement: TABLE S1 [file mBio.03264-20-st001.docx]

**Table S1. Primers for making mM1 and mM8 mutants**

| Primer Name | Primer sequence |
| --- | --- |
| mM1-RING1-F | 5’- /5Phos/ CAGGACATCTCCAGGATCTCTCACTGCGAA -3’ |
| mM1-RING1-R | 5’- /5Phos/ AGTAGATGGACAGACAGACAACCTTGACT -3’ |
| mM1-RING2-F | 5’- /5Phos/ ATCACACCCTCTCGCTCCACAGGAA -3’ |
| mM1-RING2-R | 5’- /5Phos/ GAGTGGGCTCTCTTCATCTCCTTCGCA -3’ |
| mM8-RING1-F | 5’- /5Phos/ CCAGGACATCTCCAGGATCTCCCACTG -3’ |
| mM8-RING1-R | 5’- /5Phos/ TTGGATGGTGTGACAGAAGTGCGAG -3’ |
| mM8-RING2-F | 5’- /5Phos/ CACCCCCTCTCACTCCACAGGGAGC -3’ |
| mM8-RING2-R | 5’- /5Phos/ ATCAGAGGGCTCTCGTCATCCCCT -3’ |
| mM1-DIRT-F | 5’- /5Phos/ CTCCAGATGACCACGAGC -3’ |
| mM1-DIRT-R | 5’- /5Phos/ CTCCATTATGAAGTCATACTTGCA -3’ |
| mM8-DIRT-F | 5’- /5Phos/ TTGCAGATGACTGCCAGT -3’ |
| mM8-DIRT-R | 5’- /5Phos/ CTCCATGATGAACTCGTACTTG -3’ |
| mM1-YA1-F | 5’- /5Phos/ GCCCAACTGTGGCGCC -3’ |
| mM1-YA1-R | 5’- /5Phos/ GGCGACTTTACACTGTACATACAT -3’ |
| mM1-YA2-F | 5’- /5Phos/ CTGAAGGCCGCCGCCCGTGT -3’ |
| mM1-YA2-R | 5’- /5Phos/ CCGGCGCCACAGTTGGACGTAGACTTTAC -3’ |
| mM8-YA1-F | 5’- /5Phos/ GCACAGTTATGGAAAAGACTCA -3’ |
| mM8-YA1-R | 5’- /5Phos/ GGCCACCTTGCACTGAACA -3’ |
| mM8-YA2-F | 5’- /5Phos/ GCTAGAGTGATCTATGTTCAGA -3’ |
| mM8-YA2-R | 5’- /5Phos/ GGCAGCCTTGAGTCTTTTCC -3’ |
| mM1-VQNC-F | 5’- /5Phos/ TGTGATCTTTGCGGCGAATTGCCCA -3’ |
| mM1-VQNC-R | 5’- /5Phos/ CGGTTGTAGGCCTTCAGCCGG -3’ |
| mM8-VQNC-F | 5’- /5Phos/ GTGATCTATGCTGCGAACTGTCCA -3’ |
| mM8-VQNC-R | 5’- /5Phos/ TCTATTGTAAGCCTTGAGTCTTTTCC -3’ |
| mM3TM1-mM1-F | 5’- AGGAGGAAACTGTTTGGAGATATGGTGTGC -3’ |
| mM3TM1-mM1-R | 5’- GATCTATCAACAGCCAGCCTGAGATGGTAG -3’ |
| mM3TM2-mM1-F | 5’- TGGAATGGCCACTGGAGGCCGTCGGACTC -3’ |
| mM3TM2-mM1-R | 5’- GACTTTACACTGTGTCCAGAAGAGGTAAATGGTG -3’ |
| mM1V-mM3TM1-F | 5’- ATCTCAGGCTGGCTGTTGATAGATCGGACAGCGGA -3’ |
| mM1V-mM3TM1-R | 5’- CACCATATCTCCAAACAGTTTCCTCCTTTCGCTCGTGGTCATC -3’ |
| mM1V-mM3TM2-F | 5’- CTCTTCTGGACACAGTGTAAAGTCTACGTC -3’ |
| mM1V-mM3TM2-R | 5’- GACGGCCTCCAGTGGCCATTCCAGCACACCATTGTC -3’ |
| mM4TM1-mM8-F | 5’- AGCGCAGGAAGATCATGTTGGGTTCTCTCTTCCTCAT -3’ |
| mM4TM1-mM8-R | 5’- TCTGCTGTGCGGTCTGAGGGACTGAAGGTCGA -3’ |
| mM4TM2-mM8-F | 5’- ATCCTAGAGTGGCCTCTCTTCCAGATCTGCTATGG -3’ |
| mM4TM2-mM8-R | 5’- GTACACCTTGCACTGGATGATGAGGCCTATGCAC -3’ |
| mM8V-mM4TM1-F | 5’- ACCTTCAGTCCCTCAGACCGCACAGCAGAGGAAATC -3’ |
| mM8V-mM4TM1-R | 5’- GGAAGAGAGAACCCAACATGATCTTCCTGCGCTCAC -3’ |
| mM8V-mM4TM2-F | 5’- CATAGGCCTCATCATCCAGTGCAAGGTGTACCTACAG -3’ |
| mM8V-mM4TM2-R | 5’- CATAGGCCTCATCATCCAGTGCAAGGTGTACCTACAG -3’ |
