# Supplementary material for: Elucidating the Antiviral Mechanism of Different MARCH Factors
Source: mBio. 2021 Mar 2;12(2):e03264-20. doi: 10.1128/mBio.03264-20 (PMC8092282; doi:10.1128/mBio.03264-20)
Supplement: TABLE S2 [file mBio.03264-20-st002.docx]

**Table S2. Primers for cloning of viral envelopes**

| Primer name | Primer sequence |
| --- | --- |
| JuninGP-F | 5’- GGGGATCCGAAATGGGGCAATTCATCAGCTTCATG -3’ |
| JuninGP-R | 5’- GGCTCGAGTTACTTGTCGTCATCGTCTTTGTAGTCGTGTCCTCTACGCC -3’ |
| LassaGP-F | 5’- GGGGATCCGAAATGGGACAAATAGTGACATTCTTCC -3’ |
| LassaGP-R | 5’- CCTCTAGATCACTTGTCGTCATCGTCTTTGTAGTCTCTCTTCCATTTCACAG -3’ |
| HPIA1-HN-F | 5’- GGAAGCTTATGGCTGAAAAAGGGA -3’ |
| HPIA1-HN-R | 5’- CCCTCTAGAGGAGATGTGACTTTACATATT -3’ |
| IAV-HA-F | 5’- GGGGATCCATGAAGGCAAACCTACTG -3’ |
| IAV-HA-R | 5’- CCCTCTAGACCGATGCATATTCTGCACTGCA -3’ |
| MV-HN-F | 5’- GGAAGCTTATGTCACCACAACGAGAC -3’ |
| MV-HN-R | 5’- GGGATATCTCTGCGATTGGTTCCATCTTC -3’ |
| CHIKV-F | 5’- GGGATCCATGGAGTTCATCC -3’ |
| CHIKV-R | 5’- GTCTAGAGTGCCTGCTGAACGACAC -3’ |
| SARS-CoV2-M-F | 5’- ATGGCAGATTCCAACGGTAC -3’ |
| SARS-CoV2-M-R | 5’- CTGTACAAGCAAAGCAATATTGTCACTG -3’ |
| CCGP1-F | 5’- GGTGGAATTGCCCTTGCCATGCATATATCATTAATGTATGCAATCC-3’ |
| CCGP1-R | 5’- CTGCCCAGTCTTTGGTCTAC -3’ |
| CCGP2-F | 5’- CAAAGACTGGGCAGTGAACTAG -3’ |
| CCGP2-R | 5’- GAGTACATCTGAGACAAGTC -3’ |
| CCGP3-F | 5’- GTCTCAGATGTACTCTCCTGTC -3’ |
| CCGP3-R | 5’- TGCAGAATTGCCCTTGCCAATGTGTGTTTTTGTAG ​-3’ |
| pcDNA-CC-F | 5’- AAAACACACATTGGCAAGGGCAATTCTGCAGATATCC -3’ |
| pcDNA-CC-R | 5’- TGATATATGCATGGCAAGGGCAATTCCACCACACT -3’ |
